# Return to work for health professionals with breast cancer as health recipients: A systematized review

**DOI:** 10.1177/10519815251410109

**Published:** 2026-02-16

**Authors:** Karen Belkić, Brigitte Wilczek

**Affiliations:** 1 Department of Oncology/Pathology, Karolinska Institute, Stockholm, Sweden; 2 Department of Medical Radiation Physics and Nuclear Medicine, Karolinska University Hospital, Stockholm, Sweden; 3 School of Community and Global Health, Claremont Graduate University, Claremont, California, USA; 4 Institute for Health Promotion and Disease Prevention Research, University of Southern California School of Medicine, Los Angeles, California, USA; 5 Department of Mammography, Karolinska University Hospital, Stockholm, Sweden

**Keywords:** breast neoplasms, rehabilitation, health personnel, occupational stress, night shift work, stress disorder, post-traumatic

## Abstract

**Background:**

Health professionals are at increased breast cancer(BC) risk. Occupational factors are likely contributory, especially nightwork. Return to work for women with BC has received much attention. However, systematic review of return-to-work among health professionals with BC is lacking.

**Objective:**

To perform systematized review of the return-to-work literature on health professionals with BC.

**Methods:**

PRISMA and ENTREQ guidelines were followed, searching PUBMED, CINAHL, PsycINFO and Web-of-Science.

**Results:**

From 2242 publications, 33 primarily qualitative studies addressed return-to-work among health professionals with BC. Fourteen return-to-work studies included some health professionals with BC. Ten studies addressed return-to-work among health professionals with cancer; 264 of whom had BC. Of nine case-studies/self-reports of health professionals with BC, seven worked within oncology. Occasionally-mentioned baseline working conditions included long workhours, nightshifts and busy schedules/multi-tasking. Particular concerns regarding chemotherapy for health professionals were infection risk, fatigue, cognitive function and appearance, the latter often impacting BC disclosure to patients. Emotional burdens when confronting patients’ health problems while afflicted with BC were highlighted. Occasionally-implemented modifications with return-to-work were shortened workhours, nightwork elimination, modified duties or job change. Salutogenic developments with return-to-work included emotional rewards: feeling needed and enhanced sensitivity/empathy for patients with cancer. Issues surrounding the initial BC diagnosis were very delicate for health professionals. Three oncology nurses with BC were diagnosed with post-traumatic stress disorder.

**Conclusions:**

Much more attention should be directed to the occupational needs as well as potential contributions of health professionals with BC. Participatory action research should guide intervention studies aimed at identifying the healthiest RTW options for this special cohort.

## Introduction

Health professionals are at increased risk of breast cancer (BC).^1,2^ A number of occupational exposures may heighten their BC risk. Among these are ionizing radiation and nightshift work; for example, investigations conducted primarily among nurses indicate a significant association between prolonged exposure to nightshift work and BC risk.^3,4^ For nurses who were overweight or had work-related effort reward imbalance (ERI), years of nightshift work were linked to increased age acceleration.^5^ The latter has been related to elevated BC risk.^6^ Compared to nurses working only day shifts, those who permanently worked nightshifts were found to have shorter sleep duration and greater sleep debt.^7^

The emotional burden experienced by health professionals, as well as ERI, deleteriously impact sleep.^8^ Among physicians, the total work stressor burden, as assessed by the Occupational Stressor Index (OSI), has been significantly associated with poor overall sleep quality (Pittsburgh Sleep Quality Index ≥ 6).^9^ There is some evidence that short sleep duration, as well as poor or irregular sleep, are linked to heightened BC risk.^10,11^

As the most frequently-diagnosed malignancy among women worldwide,^12^ BC is often detected and treated during paid-employment years.^13^ Overall, continued employment appears to favorably impact well-being among women who have been diagnosed with BC.^14–16^ Return to work (RTW) for women with BC has received much attention.^13–30^

Among the abiding themes that arise in relation to RTW for women with BC is the apparent benefit of implementing at least one modification in working conditions, with decrease with work hours being most frequent.^21^ On the other hand, heavy self-rated workload is reportedly a negative predictor of RTW during BC treatment.^18^ Knowledge regarding employment rights and entitlements, as well as social support have been emphasized as important for women with BC.^16,^^24–26^ Cognitive limitations, fatigue and changes in appearance are particularly important RTW issues during and after BC treatment.^13,24,25,31^ A pilot randomized controlled trial from Scotland indicates that case management vocational rehabilitation was associated with 53 fewer days of sick leave during the first 6 months after BC surgery for seven women compared to 11 women who did not receive this service.^17^ In studies among patients treated for BC as well as other malignancies, the importance of supervisors’ support for promoting sustainable, satisfying RTW experiences has been demonstrated.^32^

Notwithstanding helpful insights such as these, Porro and colleagues^33^ emphasize the need for “a better understanding of the RTW process…in order to propose appropriate interventions aimed at facilitating the RTW of [survivors of BC] and its sustainability” (p. 591). According to the Expert Panel tackling this question, a detailed consideration of the work environment, including professional status is essential.^33^ In light of their heightened risk, the question arises: What is the state of the knowledge regarding RTW among health professionals with BC? We aim herein to systematically address this question. This becomes a particularly urgent priority given the stressful work conditions faced by health professionals.

## Methods

The Preferred Reporting Items for Systematic reviews and Meta-Analyses literature search extension (PRISMA-S)^34^ guidelines were followed, with further insights gleaned regarding qualitative studies via ENTREQ (Enhancing transparency in reporting the synthesis of qualitative research)^35^ and regarding systematized review via Ref.^36^ Ethics committee approval was not required since the present study is a systematized review of published material.

### Eligibility criteria

Studies addressing RTW among women with BC were potentially eligible, insofar as health professionals of any profile were explicitly included. Breast cancer could have been at any stage and with any treatment. Return-to-work needed to have been addressed in some way in the publication. There was no restriction as to research design; narrative studies, including case- or self-reports were considered. Only full length publications (journal articles or books) were included, with no limitations regarding date of publication nor language.

### Search strategy

Comprehensive searches of the literature were carried out between mid-December 2024 and mid-March 2025 by the first author, with replication by the second author. The authors consulted librarian experts in systematic reviews at their institutions regarding the choice of search terms and strategy. The data bases used for the searches were: PUBMED, CINAHL, PsycINFO and Web of Science. Three search strategies were employed:

1. (Physicians OR Doctors OR Clinicians OR Nurses OR Physical Therapists OR Occupational Therapists OR Midwives OR Respiratory Therapists OR Health Professionals) AND (breast cancer) AND (self-disclosure) AND (work)
2. (Physicians OR Doctors OR Clinicians OR Nurses OR Physical Therapists OR Occupational Therapists OR Midwives OR Respiratory Therapists OR Health Professionals) AND (breast cancer) AND (return to work)
3. (Physicians OR Doctors OR Clinicians OR Nurses OR Physical Therapists OR Occupational Therapists OR Midwives OR Respiratory Therapists OR Health Professionals) AND (breast cancer) AND (work conditions)

The authors’ own reference lists and other sources, including within the identified references, were also examined.

### Screening procedure/inclusion

All procedures were performed directly by the authors without any extrinsic software. After omission of duplicates, the title and then abstract of each reference were examined to assess whether or not BC and RTW were likely to have been addressed. After excluding studies that did not fulfill these criteria, the entire full text of each reference was scrutinized. This procedure was detailed, multi-faceted and iterative. The key was to identify studies in which health professionals were explicitly included as patients with BC, i.e., not only care providers. The entire full text of each reference was examined independently, at least twice. Next, the empirical publications on BC and RTW that included health professionals as patients were further scrutinized at least twice. At that step, the question was whether or not there was actual information about the included health professionals. Studies were excluded insofar as work-related information (other than the most basic, such as job category or job title) was lacking about the health professionals.

### Approach to evaluating the included studies

The focal point for all the included studies was to glean as much information and insight as possible about the health professionals with BC, emphasizing work-related issues, within a clinical framework. This too was an iterative process, by which three study categories were first distinguished. These were: (1) Publications addressing RTW after BC in which some of the participants were health professionals, (2) Publications addressing RTW among health professionals with BC or other malignancies and (3) Case reports/ self-reports of health professionals with BC.

For all three study categories the following data were extracted. Firstly, publication year and country in which the investigation was performed were recorded. Next, the available oncologic data were compiled. Baseline work conditions/job stressors were reviewed when available, followed by issues of direct relevance to RTW. Then the actual data regarding RTW, especially the timing vis-à-vis initial BC diagnosis were noted. In relation to RTW, it was also noted whether or not the health professional(s) disclosed their BC diagnosis. Issues related to disclosure (or non-disclosure) were indicated, as well. Next, from the actual description, and sometimes inferentially from the narratives, we strove to assess the job stressor status with RTW, including eventual modifications in work conditions. Psychological and lifestyle issues were also evaluated as they related to BC diagnosis, treatment and rehabilitation. Further comments were made about each of the studies. The latter emphasized overriding conclusions, underscoring both the strengths as well as methodologic challenges.

Some of the steps in the data extraction procedure required adaptation according to the study category. The study design was indicated for the 1^st^ two study categories. Self-reports versus case reports were distinguished for category 3. For reports in which there were participants other than health professionals with BC (category 1), further salient findings for the entire study group were identified. For category 2, relevant insights from health professionals with other malignancies were noted. Information regarding initial BC detection were available for categories 2 and 3. Self-treatment was described in some of the Category 2 studies of health professionals. Information about how the initial BC diagnosis was conveyed was available only among some of the self-reports (Category 3).

The authors independently evaluated each of the included studies. Areas of disagreement were resolved by in-depth discussion.

## Results

**Figure 1.**
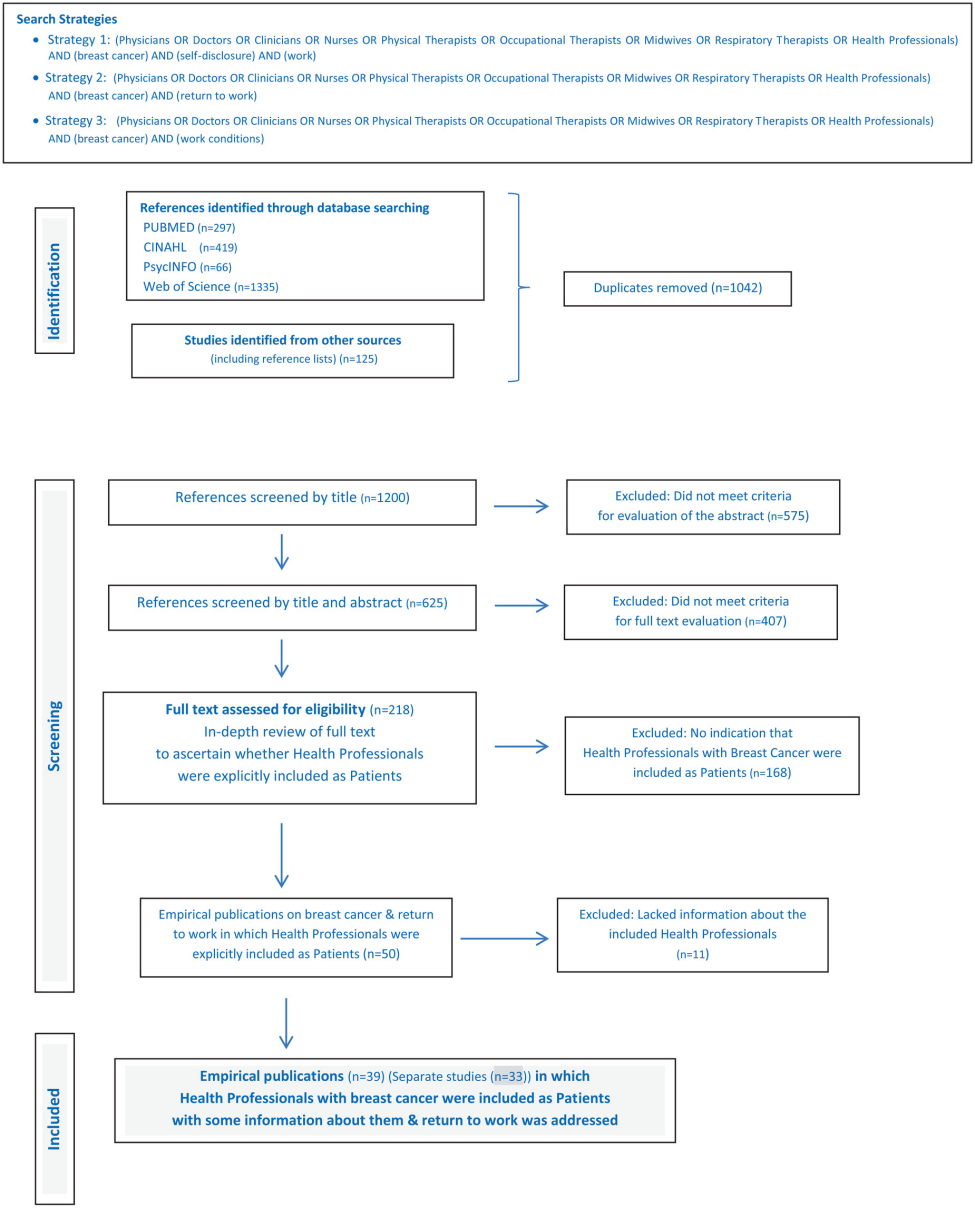
PRISMA flow chart for selection of studies addressing return to work among health professionals with breast cancer.

As summarized in the flow chart, Figure 1, altogether 2242 publications were identified via the above-described search strategies. After removing 1042 duplicates, 1200 references were screened by title, 625 of which were deemed sufficiently relevant to review the abstract. From these abstracts, 218 references were found in which BC and RTW were addressed and whose full text was then reviewed. These are listed in alphabetical order in the Supplement. Fifty empirical publications were identified in which health professionals with BC were included as patients and RTW issues were addressed. Eleven of the 50 publications lacked any further information about the health professionals and were excluded. Each of the 11 excluded papers is marked in the Supplement.

Altogether 39 publications from 33 separate studies were identified in which RTW among health professionals with BC were explicitly included, with at least some information about the health professionals and their work-related issues. These are divided into three tables, each arranged in chronological order. Table 1 contains publications in which health professionals were participants in a larger study addressing RTW after BC. Publications explicitly focusing on health professionals with cancer including BC, are presented Table 2, while case studies and self-reports of health professionals with BC are in Table 3.

**Table 1.** Studies of return to work for patients with breast cancer in which health professionals are explicitly included among the care recipients.

1^st^ Author, pub y, country	Participants, Study design	Oncologic clinical status	Baseline job conditions/stressors	Issues directly relevant to RTW	RTW//Disclosure	Work stressor status/modification with RTW	Ψ/lifestyle issues	Further comments
Kennedy et al. 2007 **UK**^37^	Three Nurses Pp#1: Age 58 ∼∼∼∼∼∼∼∼∼∼ Pp #4: Age 58 ∼∼∼∼∼∼∼∼∼∼ Pp #12: Age 64 ∼∼∼∼∼∼∼∼∼∼ ∼∼∼∼∼∼∼∼∼∼ Entire group: 29 participants, 27 female Age $\bar{x}$ 53 y **Semi-structured interviews/** **focus groups**	Pp#1 BC: Lx, RT ∼∼∼∼∼∼∼∼∼∼ Pp#4 BC: Lx, CTx, RT ∼∼∼∼∼∼∼∼∼∼ Pp#12 BC: Mx, CTx, RT, HT ∼∼∼∼∼∼∼∼∼∼ ∼∼∼∼∼∼∼∼∼∼ Entire group: CA dx within 10y 24 (83%) BC: 18 CTx —— 2 Gyn Ca 2 NHL 1 larynx	Not described	∼∼∼∼∼∼∼∼∼∼ Pp#4: Health professionals never asked her whether she would be exposed to infection at work. She felt overwhelmed by being faced with other people's health problems. Compares her situation to a receptionist (presumably also with BC) for whom RTW was beneficial	Pp#1: 3 wk ∼∼∼∼∼∼∼∼∼∼ Pp#4: 11 M ∼∼∼∼∼∼∼∼∼∼ Pp#12: 3M ∼∼∼∼∼∼∼∼∼∼ ∼∼∼∼∼∼∼∼∼∼ All with BC: From 3 wk to 18 M, some part-time, one retired **//** Entire group: All disclosed to employer ∝ needing time off for Tx	Not described specifically for the Nurses ∼∼∼∼∼∼∼∼∼∼ ∼∼∼∼∼∼∼∼∼∼ Entire group: Most described some modifications: Δ WH &/or tasks, ↓ workload	No specifics for the Nurses ∼∼∼∼∼∼∼∼∼∼ ∼∼∼∼∼∼∼∼∼∼ Entire group: Some explicitly reevaluated their priorities.	There was a wide range of RTW outcomes for the entire group of participants ∝ multifactorial considerations The Authors emphasize the role of health professionals as well as employers to facilitate successful RTW
Johnsson et al. 2010, **Sweden** ^22^	Physiotherapists: Ameli: Age 45 ∼∼∼∼∼∼∼∼∼∼ Anita: Age 51 ∼∼∼∼∼∼∼∼∼∼ Nurse Sussi: Age 46 ∼∼∼∼∼∼∼∼∼∼ ∼∼∼∼∼∼∼∼∼∼ Entire group: 16 women, age 44–58y **Narrative**	Ameli: HT ∼∼∼∼∼∼∼∼∼∼ Anita: HT + RT ∼∼∼∼∼∼∼∼∼∼ Sussi: CTx, HT ∼∼∼∼∼∼∼∼∼∼ ∼∼∼∼∼∼∼∼∼∼ Entire group: All BC, surgical Tx, recurrence free Seven: CTx Seven: RT Twelve: HT	Not described ∼∼∼∼∼∼∼∼∼∼ ∼∼∼∼∼∼∼∼∼∼ Entire group: Various occupations: Two teachers, a cook, a controller, a social worker, a hairdresser, controller, a train driver, a bank clerk, a shop assistant, a marketing manager, one self-employed, one unemployed	∼∼∼∼∼∼∼∼∼∼ Sussi: Opposition from employer for RTW ∼∼∼∼∼∼∼∼∼∼ ∼∼∼∼∼∼∼∼∼∼ Entire group: considered “belonging to the labour market …health promoting” (p.319) -Those on sick leave at 12 M reported opposition from employers for attempting RTW -Six of the seven who received CTx: on sick leave at 12M	$\bar{p}$ 1y: Ameli: Yes ∼∼∼∼∼∼∼∼∼∼ Anita: Yes ∼∼∼∼∼∼∼∼∼∼ Sussi: No ∼∼∼∼∼∼∼∼∼∼ ∼∼∼∼∼∼∼∼∼∼ Entire group: 50% yes **//** Entire group: Disclosure not explicitly addressed	Ameli: “told how her life had been normalized through [RTW]: ‘I started to work part-time and then I increased the time. It was so pleasant to work because it was so normal.’” (p.320)		Work as physiotherapists might have been a promoting factor for RTW Further clinical details not given, but for the health professionals & for the entire group, BC severity reflected in the need for CTx could have impacted RTW
						∼∼∼∼∼∼∼∼∼∼ ∼∼∼∼∼∼∼∼∼∼ Entire group: “Narratives emphasized the importance of social support received from employers and coworkers during the process of RTW” (p.320)	∼∼∼∼∼∼∼∼∼∼ ∼∼∼∼∼∼∼∼∼∼ Entire group: Living arrangements similar for those at 12 M who had RTW versus those on sick leave	
Munir et al. 2010 **UK**^38^	One Medical Secretary/ Counsellor Pp10: Age 48 ∼∼∼∼∼∼∼∼∼∼ ∼∼∼∼∼∼∼∼∼∼ Entire group: 13 women age 36–60, all employed at dx **Semi-structured interviews/** **focus groups**	Pp10: BC: Surgical Tx, CTx, RT, immunoTx ∼∼∼∼∼∼∼∼∼∼ ∼∼∼∼∼∼∼∼∼∼ Entire group: All 13 BC + CTx 12 surgery, 12 RT, 6 HT, 2 immunoTx No other chronic illness	Pp10: “High stress” (p.1366) but memory was reportedly good & could perform required multitasking/part-time ∼∼∼∼∼∼∼∼∼∼ ∼∼∼∼∼∼∼∼∼∼ Entire group: Various occupations: Clerical assistant, department head, accountant, hair stylist, administrator, shop assistant, standards officer, director, Two managers, two warehouse employees Seven full time, six part-time	Pp10: Notes CTx impact on memory, “cannot deal with too much stress anymore” (p.1366) ∼∼∼∼∼∼∼∼∼∼ ∼∼∼∼∼∼∼∼∼∼ Entire group: RTW ∝ cognitive ability $\bar{p}$ CTx, awareness of cognitive failures, subsequent impact on confidence in fulfilling job tasks.	Pp10: >4 wk sick leave ∼∼∼∼∼∼∼∼∼∼ ∼∼∼∼∼∼∼∼∼∼ Entire group: 85%: >4 wk sick leave // Entire group: Disclosure not explicitly addressed but implicit in some cases	Pp10: ↓ WH, 3 h/wk ∼∼∼∼∼∼∼∼∼∼ ∼∼∼∼∼∼∼∼∼∼ Entire group: Seven no Δ, four stopped working/retired, two ↓ WH Support from employer/coworkers promoted confidence in fulfilling work tasks	Pp10: Describes “switch[ing] off” ∼∼∼∼∼∼∼∼∼∼ ∼∼∼∼∼∼∼∼∼∼ Entire group: Some reports of CTx cognitive impact on activity outside work ∝ decision-making, or > 1 person speaking	The Authors suggest that promoting self-efficacy & training in cognitive compensatory strategies could be examined as interventions especially important to bolster work ability in the face of the cognitive side-effects of CTx
Tighe et al. 2011 **UK**^23^	One nurse: Anne Age 43 y ∼∼∼∼∼∼∼∼∼∼ ∼∼∼∼∼∼∼∼∼∼ Entire group: 10 women all newly dx BC, age $\bar{x}=51\;y)$ **Thematic narrative interviews**	Anne: CTx + RT (Surgical Tx likely but not explicitly noted) ∼∼∼∼∼∼∼∼∼∼ ∼∼∼∼∼∼∼∼∼∼ Entire group: –Eight: surgical Tx –Four: CTx + RT –Eight: Grade 1 or 2 –Three: HT + RT	Anne: Worked 70–80 h/wk, evenings, on call, early AM meetings ∼∼∼∼∼∼∼∼∼∼ ∼∼∼∼∼∼∼∼∼∼ Entire group: –Four: professional full-time work –Two: part-time work –Two: retired	Anne: Immediately post BC dx: Expressed the need to “completely cut off work”. After CTx completion: (3 M later) identified long WH & on call as key stressors ∼∼∼∼∼∼∼∼∼∼ ∼∼∼∼∼∼∼∼∼∼ Entire group: –Disturbed sleep/nightmares described by one woman –Fatigue –Lymphedema	Anne: Yes $\bar{p}$ 1y // Implicit disclosure at work ∼∼∼∼∼∼∼∼∼∼ ∼∼∼∼∼∼∼∼∼∼ Entire group: –Sick leave ∝ lymphedema described by one woman // Disclosure not explicitly discussed elsewhere	Anne: At 3^rd^ interview (6 M $\bar{p}$ dx): “Recognize[s]…as a nurse I can learn from is being a patient. I have learnt so much about change, I need to make some changes to improve patients’ lives and journeys” (p.230) At 4^th^ interview ( $\bar{p}$ 1y): “Work[s] less, live[s] more…very clear…about boundaries and that I am having a break at lunchtime. I do have to be on call but equally I insist that the reason for ringing is necessary. I don’t think about work when I get home” (p.230) ∼∼∼∼∼∼∼∼∼∼ ∼∼∼∼∼∼∼∼∼∼ Entire group (individual narratives): Impact of BC on relationships with co-workers, as well as with family, friends and health professionals—This, in turn, influences coping strategies and how to “make sense of their [BC] experience” (p. 226) One participant: Clinical Depression diagnosed $\bar{p}$ 1y Impact of CTx on appearance: “soul-destroying”, with regrowth of hair: feeling “more like normal self”(p.228)		Anne as a nurse indicates that with RTW by setting boundaries & not thinking about work when at home, she has diminished intrinsic ERI. Nine women explicitly declined participation. Cited reasons included avoiding being reminded of the cancer. Thus, the low participation rate may reflect, at least in part, selection bias.
Tamminga et al. 2012 **NL** ^24^	Nurse Specialist: Age 47 at dx, 50 at interview (Participant B) ∼∼∼∼∼∼∼∼∼∼ ∼∼∼∼∼∼∼∼∼∼ Entire group: 12 participants, age 28–47 at BC dx, 31–51 at interview All BC **Semi-structured interviews**	Nurse Specialist: Surgical Tx, CTx, RT, Tx lasted 7M ∼∼∼∼∼∼∼∼∼∼ ∼∼∼∼∼∼∼∼∼∼ Entire group: All Surgical Tx Ten: CTx Nine: RT Six: HT Three: Herceptin Two: Ovariectomy Two: MET	Nurse Specialist: Permanent employment, 30 y work history, 32 h/wk, shift work ∼∼∼∼∼∼∼∼∼∼ ∼∼∼∼∼∼∼∼∼∼ Entire group: -Various occupations: Five managers, a call center supervisor, a coach, a senior policy officer, a taxi driver, a communication specialist -Nine permanent, -Two temporary employment -0ne freelance -Work hx: 0.5–12 y -WH: 10–40 h/wk -Shift work: Four	Nurse Specialist: Early after RTW, cognitive SE ∝ could not recall login code for accessing medication ∼∼∼∼∼∼∼∼∼∼ ∼∼∼∼∼∼∼∼∼∼ Entire group: -75% faced “many barriers in their RTW process” (p.147) -RTW facilitated by lack of long-term SE – RTW impeded by: Lymphedema, other comorbidity, ↓ concentration	Nurse Specialist: Partial disability pension, 50% RTW ∼∼∼∼∼∼∼∼∼∼ ∼∼∼∼∼∼∼∼∼∼ Entire group: “different complex pattern of RTW” (p.146) // All disclosed to supervisor and colleagues	Nurse Specialist: Worsened WH & work content, no shift work New sick leave episode ∼∼∼∼∼∼∼∼∼∼ ∼∼∼∼∼∼∼∼∼∼ Entire Group: -RTW facilitators: Job content & control, Performing less demanding tasks, Support from work environment, Flexible WH, Ease of taking time off -WH: 10–40 h/wk -Five: Worsened WH -Two: Shift work	No specifics for the Nurse Specialist ∼∼∼∼∼∼∼∼∼∼ ∼∼∼∼∼∼∼∼∼∼ Entire Group: confidence, energy, support outside work: RTW facilitators	The Authors recommend that interventions for survivors of BC be aimed at barriers & facilitators of RTW at various points in time. The gender of the participants not explicitly stated.
Groeneveld et al. 2013 **NL**^39^	P3: Senior nurse, age 49 ∼∼∼∼∼∼∼∼∼∼∼∼∼∼ P7: Nurse, age 48 ∼∼∼∼∼∼∼∼∼∼ ∼∼∼∼∼∼∼∼∼∼ Entire group: Ten participants Nine female Age 39–58 70% BC **Semi-structured interviews** $\bar{p}$ **Supervised Exercise Program**	Senior Nurse P3: BC, surgical Tx, CTx, RT ∼∼∼∼∼∼∼∼∼∼∼∼∼∼ Nurse P7: BC, surgical Tx, CTx ∼∼∼∼∼∼∼∼∼∼ ∼∼∼∼∼∼∼∼∼∼ Entire group: All CTx, 90% surgery,	No further description for the Nurses ∼∼∼∼∼∼∼∼∼∼ ∼∼∼∼∼∼∼∼∼∼ Entire group: Various occupations: A secretary, an engineer, a logistics employee, a teacher, a legal officer, a receptionist, a complaints desk employee, one without job	Nurse P3: Viewed being able to cycle ∝ RTW ∼∼∼∼∼∼∼∼∼∼∼∼∼∼ Nurse P7: described short regular meetings during which concern for her was expressed ∼∼∼∼∼∼∼∼∼∼∼∼∼∼ ∼∼∼∼∼∼∼∼∼∼∼∼∼∼ Entire group: Most considered exercise program helped RTW	Nurse P3: Yes ∼∼∼∼∼∼∼∼∼∼∼∼∼∼ Nurse P7: Yes ∼∼∼∼∼∼∼∼∼∼∼∼∼∼ ∼∼∼∼∼∼∼∼∼∼∼∼∼∼ Entire group: 80% RTW //Disclosure implicit	No further description for Nurse P3 ∼∼∼∼∼∼∼∼∼∼∼∼∼∼ Nurse P7: describes starting work earlier to be able to go directly thereafter to the exercise program. Describes dilemma of ↓ WH ∝ participation in the exercise program ∼∼∼∼∼∼∼∼∼∼∼∼∼∼ ∼∼∼∼∼∼∼∼∼∼∼∼∼∼ Entire group: Most noted cognitive impairments hampered work performance	P3: Senior nurse considers fitness after exercise program better than prior to dx, when fitness was already good ∼∼∼∼∼∼∼∼∼∼∼∼∼∼ No further description for Nurse P7 ∼∼∼∼∼∼∼∼∼∼∼∼∼∼ ∼∼∼∼∼∼∼∼∼∼∼∼∼∼ Entire group: The majority enjoyed the exercise program (cycle) which ↑ energy	For this small study with the majority treated for BC, a synergistic positive interaction is suggested between RTW & exercise
Sun et al. 2016 **U.S.**^25^	At least one HCW ∼∼∼∼∼∼∼∼∼∼ ∼∼∼∼∼∼∼∼∼∼ Entire group: 35 participants total Age: 48.1 ± 10.3y, 77% college educated All BC, female **Semi-structured interviews**	No further specifics for HCW ∼∼∼∼∼∼∼∼∼∼ ∼∼∼∼∼∼∼∼∼∼ Entire group: Surgery, CTx &/or RT, during active Tx, w/in 6 M of completing active Tx, No progressive disease or MET No previous CA dx	No further specifics for the HCW ∼∼∼∼∼∼∼∼∼∼ ∼∼∼∼∼∼∼∼∼∼ Entire group: All employed >20 h/wk at time of BC dx, 57% “professionals”	One participant described receiving phone calls from the cancer center when seeing patients/also described concern about hair loss in relation to patients ∼∼∼∼∼∼∼∼∼∼∼∼∼∼ ∼∼∼∼∼∼∼∼∼∼∼∼∼∼ Entire group: Barriers: –Symptoms –Emotional distress –Appearance Δ –Time constraints –Lack of support from supervisor &/or coworkers –Job-related health hazards, –Rigid job profile	HCW: Implicitly yes //A participant who “worked with patients” expressed her concern about disclosure to patients ∼∼∼∼∼∼∼∼∼∼∼∼∼∼ ∼∼∼∼∼∼∼∼∼∼∼∼∼∼ Entire group: All yes during active Tx, but at least one stopped subsequently //No further discussion of disclosure	–Request colleague to “step in” with challenging tasks e.g., clinical procedure ∼∼∼∼∼∼∼∼∼∼∼∼∼∼ ∼∼∼∼∼∼∼∼∼∼∼∼∼∼ Entire group: Work-related facilitators of RTW during active Tx –“Positive aspects of work”: e.g., affinity for the job, feelings of normalcy & responsibility, healthy social interactions – ↓ WH, ↓ workload – Time off taken during part of Tx – Δ work tasks (↓ physical demands) – Protected job position, paid time off – Flexible WH – Option of work by phone/remotely – Emotional support: sensing that coworkers & supervisors truly care –Aid job performance via: e.g., notes to bolster memory, asking for help, improving physical (e.g., office) conditions –Coverage by coworkers	No specifics for the HCW ∼∼∼∼∼∼∼∼∼∼∼∼∼∼ ∼∼∼∼∼∼∼∼∼∼∼∼∼∼ Entire group: Non-work facilitators of RTW during active Tx: –Positive attitude –Support outside the workplace –Exercise, healthy diet, cosmetics to enhance appearance	Important insights are provided regarding work conditions & RTW vis-à-vis BC with a few “glimpses” for HCW The Authors emphasize the need for comprehensive instructions for the women with BC, as well as for employers regarding ergonomic work place and adaptation of technology & tools to meet the women's needs & capacity.
Musti et al. 2018 **Italy**^40^	Sixty (12%) employed in “health/social service sector” ∼∼∼∼∼∼∼∼∼∼∼∼∼∼ ∼∼∼∼∼∼∼∼∼∼∼∼∼∼ Entire group: 503 participants All BC, female Age: $\bar{x}$ 51.5 ± 3.6 **Cross-sectional questionnaire-based study**	No further specifics for HCW ∼∼∼∼∼∼∼∼∼∼∼∼∼∼ ∼∼∼∼∼∼∼∼∼∼∼∼∼∼ Entire group: -All surgical Tx -32% Mx -42% Lymph node dissection -50% CTx -73% RT -76% HT -24% Breast reconstruction	No further specifics for HCW ∼∼∼∼∼∼∼∼∼∼∼∼∼∼ ∼∼∼∼∼∼∼∼∼∼∼∼∼∼ Entire group: All employed at time of BC dx 85%: Permanent contract	Thirty-three (55%) in health/social service sector: perceived ↓ work ability (2^nd^ largest % of the 8 included sectors) ∼∼∼∼∼∼∼∼∼∼∼∼∼∼ ∼∼∼∼∼∼∼∼∼∼∼∼∼∼ Entire group: 20% upper arm physiotherapy	No further specifics for HCW ∼∼∼∼∼∼∼∼∼∼∼∼∼∼ ∼∼∼∼∼∼∼∼∼∼∼∼∼∼ Entire group: -25% < 1M -46% 1 M to < 6M -24% ≥ 6M (6% missing data)/ Disclosure not mentioned	No further specifics for HCW ∼∼∼∼∼∼∼∼∼∼∼∼∼∼ ∼∼∼∼∼∼∼∼∼∼∼∼∼∼ Entire group: N = 31: Δ job Those with ↓ perceived work ability: Lack support from employer & colleagues, feeling discrimination if ↓ perceived work ability (p < 0.001). Work adjustments (52%) vs. (16% if no ↓ perceived work ability) (p < 0.001) Work adjustments: N = 66: ↓ physical effort N = 46: ↓ WH N = 21: Flexible WH N = 21: ↓ workpace N = 14: Rest breaks introduced N = 9: ↓ mental effort	No further specifics for HCW ∼∼∼∼∼∼∼∼∼∼∼∼∼∼ ∼∼∼∼∼∼∼∼∼∼∼∼∼∼ Entire group: 37% reported Ψ/marital problems prior to BC dx 20% received Ψ support	This relatively large cross-sectional study, provides valuable general information re: work adjustments for women with BC, and their relation to perceived work ability. Over half of the participants within the health/social sector perceived their work ability to have been diminished. It would have been helpful to know which work adjustments were most effective for this sector.
Zamanzadeh et al. 2018 & 2019 **Iran**^41,42^	-PN 8, 9*: Nurse, age 48 ——— -PN 11,12*: Nurse, age 36 ——— -PN 12,10*: Nurse, age 35 ——— -PN 15, 14: Nurse manager, age 49 ∼∼∼∼∼∼∼∼∼∼ ∼∼∼∼∼∼∼∼∼∼ Entire group: Age: $\bar{x}$ 40.8 ± 11y RTW & completed initial Tx: eligibility criteria for study In ^41^ Twenty participants, nine female (all those with BC) In ^42^ 19 participants eight female (all those with BC) Semi-structured interviews *ID numbers in Ref 2018 vs. 2019	-PN 8, 9: BC CTx ——— -PN 11,12: BC CTx ——— -PN 12,10: BC ——— -PN 15, 14: BC ∼∼∼∼∼∼∼∼∼∼ ∼∼∼∼∼∼∼∼∼∼ Rest of group: -Six: Leukemia -Five: CRC -Five: BC One each: -Lymphoma -Lung CA -Hepatic CA -Testicular CA	——— -PN 11,12: Implicit busy shifts, night shift work ——— ——— ∼∼∼∼∼∼∼∼∼∼ ∼∼∼∼∼∼∼∼∼∼ Rest of group: -Three workers -Two Carpet Weavers -Two self-employed One: -Nurse Aide -Physician -Teacher -Secretary -Office clerk -Barber -Mechanic -Pharmacy technician	-PN 8,9 worried that Δ appearance (alopecia, weight loss) → non-acceptance by colleagues ——— -PN 11,12 fatigue ∝ busy shifts, could not endure night shifts nor stand for too long ——— –PN 12,10: Employers & colleagues expected her to work as before. Sarcastic reaction when she could not lift a heavy book. ——— -PN 15,14: Helping her patients & contact with colleagues motivated RTW ∼∼∼∼∼∼∼∼∼∼ ∼∼∼∼∼∼∼∼∼∼ Entire group: Uncertainty & worry re: RTW—Lacked information about their illness, their abilities & whether RTW could provoke recurrence. Gender difference re: RTW meaning (males viewed RTW as vital to their self-image)	-PN 8,9: Nurse: 16 M ——— -PN 11,12: Nurse: 11M ——— -PN 12,10: Nurse: 8M ——— -PN 15,14: Nurse Manager: 9 M/Worried nausea could come while talking to someone ∼∼∼∼∼∼∼∼∼∼ ∼∼∼∼∼∼∼∼∼∼ Entire group: RTW 4 to 16 M $\bar{p}$ Dx/ Disclosure implicit, but rarely explicitly discussed	-PN 8,9 Due to pressure and “stressful environments…asked for workplace replacement.” (p.2401) Received only base salary, insufficient for expenses ——— ——— -PN 12,10: Could not work as she had done prior to BC ——— -PN 15,14: Colleagues treated her such that she did not feel sick ∼∼∼∼∼∼∼∼∼∼ ∼∼∼∼∼∼∼∼∼∼ From rest of group: -Male nurses’ aide with lymphoma was transferred to the hemodialysis ward which he considered appropriate for his condition. -Male barber with leukemia: needed to leave work from medical appointments –Female office clerk with CRC: easier work assignment from supervisor	PN 8,9 Afraid of stigmatization as disabled ——— -PN 11,12: Easily annoyed when feeling overwhelmed ∼∼∼∼∼∼∼∼∼∼ ∼∼∼∼∼∼∼∼∼∼ From rest of group: –Female office clerk with CRC: When home unemployed felt overwhelmed by thoughts of dying	For the entire group, the Authors conclude that RTW decisions vary according to the individual. They point out that employers, as well as health providers, particularly rehabilitation specialists should be prepared to adapt the work place to meet the needs of cancer survivors. The Authors strongly recommend initiating occupational rehabilitation immediately after CA dx, in conjunction with further dx procedures and Tx.
Şengün İnan et al. 2020 **Turkey**^16^	MD#1: Age 33 ∼∼∼∼∼∼∼∼∼∼∼∼∼∼ MD#2: Age 54 ∼∼∼∼∼∼∼∼∼∼∼∼∼∼ ∼∼∼∼∼∼∼∼∼∼∼∼∼∼ Entire group: 12 Age: $\bar{x}=48\;y\;(33-58)$ All female with BC **Semi-structured interviews**	MD #1: Mx, CTx, RT; 27 M $\bar{p}$ Tx ∼∼∼∼∼∼∼∼∼∼∼∼∼∼ MD #2: Breast-conserving surgery, CTx, RT, HT; 36 M $\bar{p}$ Tx ∼∼∼∼∼∼∼∼∼∼∼∼∼∼ ∼∼∼∼∼∼∼∼∼∼∼∼∼∼ Entire group: Surgery + (CTx &/or RT &/or HT)	MD#1: Employed as a Physician ∼∼∼∼∼∼∼∼∼∼∼∼∼∼ MD#2: Employed as a Medical Specialist & Assistant Professor ∼∼∼∼∼∼∼∼∼∼∼∼∼∼ ∼∼∼∼∼∼∼∼∼∼∼∼∼∼ Entire group: Civil servants, teachers, kitchen staff, lawyer, police officer	No further specifics MD#1 ∼∼∼∼∼∼∼∼∼∼∼∼∼∼ MD#2: Concern that she now “move[s] slowly” re: work performance ∼∼∼∼∼∼∼∼∼∼∼∼∼∼ ∼∼∼∼∼∼∼∼∼∼∼∼∼∼ Entire group: -Willing to RTW, but uncertain re: process especially re: symptoms, -support from colleagues important	MD#1: Full RTW ∼∼∼∼∼∼∼∼∼∼∼∼∼∼ MD#2 Full RTW // considered repeated explanations re: her BC tiresome ∼∼∼∼∼∼∼∼∼∼∼∼∼∼ ∼∼∼∼∼∼∼∼∼∼∼∼∼∼ Entire group: All full RTW ≥ 6 M, from 10–36 M $\bar{p}$ Tx// Some other participants found disclosure burdensome	No further specifics MD#1 ∼∼∼∼∼∼∼∼∼∼∼∼∼∼ MD#2: Others suggested that she would not need to give some of the courses as previously ∼∼∼∼∼∼∼∼∼∼∼∼∼∼∼∼ ∼∼∼∼∼∼∼∼∼∼∼∼∼∼∼∼ Entire group: Supportive work environment helped maintain RTW: Flexible WH, shared workload & responsibilities, lack of discrimination, time off for medical appointments, avoid pitying attitudes	MD#1: Notes becoming “more socialized” with RTW” (p. E332) ∼∼∼∼∼∼∼∼∼∼∼∼∼∼ No further specifics MD#2 ∼∼∼∼∼∼∼∼∼∼∼∼∼∼ ∼∼∼∼∼∼∼∼∼∼∼∼∼∼ Entire group: -“RTW positively influences psychosocial well-being” (p.E328) -Family support important -↑ assertiveness	This is one of the few general RTW studies in which insights are provided specifically about RTW for physicians with BC The Authors underline the salutogenic aspects of RTW for women with BC, despite attendant uncertainties
Van Maar-schalkerweerd et al. 2020 **NL**^43^	Two HCW P1.6: Age 59 ∼∼∼∼∼∼∼∼∼∼∼∼∼∼ P1.7: Age 51 ∼∼∼∼∼∼∼∼∼∼∼∼ ∼∼∼∼∼∼∼∼∼∼∼∼ Entire group: 19, All 5–10 y $\bar{p}$ BC dx, Age 39–59, All female, employed at BC dx. **Semi-structured interviews/** **focus group**	P1.6: Surgical Tx, RT ∼∼∼∼∼∼∼∼∼∼∼∼∼∼ P1.7: CTx, RT ∼∼∼∼∼∼∼∼∼∼∼∼ ∼∼∼∼∼∼∼∼∼∼∼∼ Entire group: BC with various Tx	P1.6: Worked 40 h/wk ∼∼∼∼∼∼∼∼∼∼∼∼∼∼ P1.7: Worked 25 h/wk ∼∼∼∼∼∼∼∼∼∼∼∼∼∼∼∼∼∼∼ ∼∼∼∼∼∼∼∼∼∼∼∼∼∼∼∼∼∼∼ Entire group: Worked 15–50 h/wk (13 white collar, one blue collar, three self-employed, in addition to the two HCW)	P1.6: worked during Tx, received help from colleagues ∼∼∼∼∼∼∼∼∼∼∼∼∼∼ P1.7: Notes fatigue ∼∼∼∼∼∼∼∼∼∼∼∼ ∼∼∼∼∼∼∼∼∼∼∼∼ Entire group: Good prognosis encouraged RTW	P1.6: RTW ∼∼∼∼∼∼∼∼∼∼∼∼∼∼ P1.7: RTW ∼∼∼∼∼∼∼∼∼∼ ∼∼∼∼∼∼∼∼∼∼ Entire group: 10 unemployed (some volunteers)**/** Entire group: several concealed BC dx, especially to new employer	P1.6: Worked 40 h/wk ∼∼∼∼∼∼∼∼∼∼∼∼∼∼ P1.7: Worked 32 h/wk (notes ↑ WH ∝ financial reasons, although tired) ∼∼∼∼∼∼∼∼∼∼∼∼∼∼∼∼∼ ∼∼∼∼∼∼∼∼∼∼∼∼∼∼∼∼∼ Entire group: 95% Δ employment status 5–10 y $\bar{p}$ BC dx, flexible WH	P1.6: No supportive Intervention ∼∼∼∼∼∼∼∼∼∼∼∼∼∼ P1.7: No supportive Intervention ∼∼∼∼∼∼∼∼∼∼∼∼∼∼∼∼∼ ∼∼∼∼∼∼∼∼∼∼∼∼∼∼∼∼∼ Entire group: 68% received the supportive Intervention (Exercise or Ψ education)	This study is quite unusual in the time lag 5–10 y after BC dx. Neither of the HCW received the intervention The Authors underscore the need for longer term interventions for BC survivors
Vignes et al. 2020 **France**^44^	29 in health/social work ∼∼∼∼∼∼∼∼∼∼∼∼ ∼∼∼∼∼∼∼∼∼∼∼∼ Entire group: N = 134, all female, employed Median age: 54 (IQR: 48.3–57.6) **Cross sectional study**	No further specifics for HCW ∼∼∼∼∼∼∼∼∼∼∼∼ ∼∼∼∼∼∼∼∼∼∼∼∼ Entire group: All upper limb lymphedema ∝ BC (52% DAL) 62%: Mx 99%: Axillary node excision 96%: RT 91%: CTx 75%: HT 20%: MET	–Not described	-Lymphedema + Mx: ↑ interference with professional activities ↑ impaired arm use ∝ ↑ sick leave Health professionals: Intermediate arm use: nurse, dentist High arm use: nurse's aide -41% global arm use impairment in work-related public contact	∼∼∼∼∼∼∼∼∼∼ ∼∼∼∼∼∼∼∼∼∼ Entire group: Apparently all RTW/more obliged to disclose to supervisors if DAL	∼∼∼∼∼∼∼∼∼∼∼∼ ∼∼∼∼∼∼∼∼∼∼∼∼ Entire group: –70% full time employed -Work station adaptation particularly ergonomic Δ for 27% ∝ high satisfaction rating, more frequent for women with DAL -7.5% Δ job ∝ lymphedema	–Not described	The Authors note the impact of upper limb lymphedema on work & that workstation adaptation can be helpful. No specifics re: interventions for HCW The role of occupational physicians is underscored in evaluating lymphedema-attributed difficulties to formulate & implement needed workplace modifications
Akezaki et al. 2021 **Japan**^45^	Eleven female “care workers” (15% of participants) ∼∼∼∼∼∼∼∼∼∼ ∼∼∼∼∼∼∼∼∼∼ Entire group: N = 73 Age $\bar{x}$ =52.5 ± 9.1 All female with BC **Retrospective RTW study**	No further specifics for HCW ∼∼∼∼∼∼∼∼∼∼ ∼∼∼∼∼∼∼∼∼∼ Entire group: Mx & axillary lymph node dissection 85%: CTx 36%: RT 19%: HT	No further specifics for HCW ∼∼∼∼∼∼∼∼∼∼ ∼∼∼∼∼∼∼∼∼∼ Entire group: All 73 women were working prior to surgery	No further specifics for HCW ∼∼∼∼∼∼∼∼∼∼ ∼∼∼∼∼∼∼∼∼∼ Entire group: DASH & Shoulder flexion ROM-T ∝ RTW at 3 M (p < 0.05)	Four of 11 (36%) RTW at 3 M among the care workers, lowest % RTW except for teachers ∼∼∼∼∼∼∼∼∼∼ ∼∼∼∼∼∼∼∼∼∼ Entire group: 49% RTW at 3 M/ Disclosure not mentioned	No further specifics for HCW ∼∼∼∼∼∼∼∼∼∼ ∼∼∼∼∼∼∼∼∼∼ Entire group: RTW to same workplace, with possible ↓ WH	–Not described	No further details about the 11 “care workers” in this relatively large retrospective RTW study The Authors underscore the need for postoperative rehabilitation for women with BC following axillary lymph node dissection to facilitate RTW .
Algeo et al. 2022 **Ireland**^26^	One nurse (case P9) Age: 60 ∼∼∼∼∼∼∼∼∼∼∼ One MD (case P36) Age: 39 ∼∼∼∼∼∼∼∼∼∼∼∼ ∼∼∼∼∼∼∼∼∼∼∼∼ Entire group: 15 women with BC RTW within 24 M, Age $\bar{x}=51.2$ Employed at time of dx **Narrative/** **Semi-structured Interviews**	Stage II BC Mx, HT, Hysterectomy ∼∼∼∼∼∼∼∼∼∼∼∼ Stage III BC, Neoadj CTx, Lx, Re-excision, RT, Herceptin ∼∼∼∼∼∼∼∼∼∼∼∼ ∼∼∼∼∼∼∼∼∼∼∼∼ Entire group: All 15 women 1 ° BC Stage I-III 11: CTx, 12: RT 8: Lx 8: HT	Public sector ∼∼∼∼∼∼∼∼∼∼∼∼ Public sector ∼∼∼∼∼∼∼∼∼∼∼∼∼∼∼∼∼∼∼ ∼∼∼∼∼∼∼∼∼∼∼∼∼∼∼∼∼∼∼ Entire group: All 15 women with BC had been working at time of dx.	None mentioned ∼∼∼∼∼∼∼∼∼∼∼∼∼ Income insurance alleviated financial stress prior to RTW ∼∼∼∼∼∼∼∼∼∼∼∼- ∼∼∼∼∼∼∼∼∼∼∼∼ Entire group: Most were unaware of employment rights -Concern re: ↓ pay with sick leave (though some had full pay), others used annual leave or holiday time	$\bar{\;p}$ 10 M/ Not reported ∼∼∼∼∼∼∼∼∼∼ $\bar{\;p}$ 16 M/Yes (apparently) ∼∼∼∼∼∼∼∼∼∼ ∼∼∼∼∼∼∼∼∼∼ Entire group: All 15 women took some time from work: $\bar{x}$ =14 Wk / Disclosure implied, but not an explicit theme	Not reported ∼∼∼∼∼∼∼∼∼∼∼∼∼∼∼∼∼ ∼∼∼∼∼∼∼∼∼∼∼∼∼∼∼ -Direct discrimination, recalling resistance by colleagues regarding promotion She suggested WH limit, which was deemed too low by the Occupational Health Service ∼∼∼∼∼∼∼∼∼∼∼∼∼∼∼∼∼∼∼∼∼∼∼∼∼∼ ∼∼∼∼∼∼∼∼∼∼∼∼∼∼∼∼∼∼∼∼∼∼∼∼∼∼ ∼∼∼∼∼∼∼∼∼∼∼∼ Entire group: -Most did not note discrimination on RTW -Those who knew about rights knew that this included “reasonable accommodations” -Various Δwork practices–Examples: Flexible scheduling, special shoes to accommodate standing, delegating tasks to others, ergonomic adaptation (lower shelves), ↓ physical work/lifting, gradual RTW –Social support sometimes short-lived	Not reported ∼∼∼∼∼∼∼∼∼∼∼∼∼ Not reported ∼∼∼∼∼∼∼∼∼∼∼∼∼∼∼∼∼∼ ∼∼∼∼∼∼∼∼∼∼∼∼∼∼∼∼∼∼ Entire group: -One woman described feeling “fragile”	This is one of the few general RTW studies in which insights are provided specifically re: RTW for a physician with BC This study highlights the importance of legal aspects/protection for women with BC vis-à-vis RTW

BC = breast cancer, CA = cancer, CRC = colorectal cancer, CTx = chemotherapy, D = day(s), DAL = dominant arm lymphedema, DASH = Disabilities of the Arm, Shoulder and Hand, dx = diagnosis/diagnosed, ERI = effort reward imbalance, Gyn = gynecologic, h = hours, HCW = Health Care Worker, HT = hormonal therapy, hx = history, immunoTx = immunotherapy, IQR = interquartile range, Lx = lumpectomy, M = month(s), MD = physician, MET = metastatic disease, Mx = mastectomy, Neoadj = neoadjuvant, NHL = non-Hodgkin's lymphoma, NL = Netherlands, Pp = participant, pub = publication, ROM-T = range of motion test, RT = radiation therapy, RTW = return to work, SE = side effects, Tx = treatment/treating, UK = United Kingdom, U.S. = United States of America, WH = work hours, wk = week(s), y = year(s), **Ψ** = psychological, $\bar{p}=$ post (after) ∝ = in relation to, $\bar{x}$ mean, Δ = change.

**Table 2.** Studies of return to work among health professionals with breast cancer or other malignancies.

1^st^ Author, pub y, country	Participants, Study design	Oncologic clinical status	Initial &/or self Dx or Tx	Baseline job conditions/stressors	Issues directly relevant to RTW	RTW// Disclosure	Work stressor status/ Δ with RTW	Ψ/ Lifestyle issues	Further comments
DeMarco et al; Picard et al, 2004, **U.S.**^46,27^	16 nurses with BC ∼∼∼∼∼∼∼∼∼∼ ∼∼∼∼∼∼∼∼∼∼ Entire group: 25 Registered nurses with CA 23 female, recruited via postings to major teaching hospitals in northeast U.S. Age: 33 to 66, > 46: 17 **Narrative**	-One nurse intervened with a research protocol related to her own experience of having had 12 breast biopsies without adequate pain management. -One nurse volunteered to present to women contemplating surgery after having experienced breast reconstruction. This was viewed as increasing the comfort of patients $\bar{p}$ surgery. She expressed good feelings when having done so. No further specifics about the 16 nurses with BC							Albeit limited information about the nurses with BC, insights from their own experience with BC diagnosis & treatment motivated a proactive approach to subsequent clinical/research activity. The Authors note that due to self-selection, the participants may not be representative of nurses who have survived cancer. The CA Dx of the two male participants not stated
		Entire group							
		Besides BC: One nurse each with CA (of): -Esophagus -Colon -Lung -Ovary -Parotid gland -Hodgkin's lymphoma -Chronic myelogenous leukemia -Sarcoma -Melanoma 80% CA Dx w/in 5y	–An expert Onc nurse described mixing her own CTx. Later considered that a possible mistake	Various healthcare settings…including EM, ICU & oncology	-Fatigue, nausea, pain & discomfort during Tx hampered RTW	After surgery, RTW by the majority during Tx// Many disclosed Dx, a decision which they wanted to make on their own. For others, difficult to talk about the illness with coworkers	–Mainly noted having received social support from coworkers –↑ compassion for patients & colleagues –Many ↑ activity in planning & policies regarding patient care	–Most described ↑ sense of vulnerability ∝ CA Dx –Challenge of balancing work/home/Tx - Helpful/ Complementary integrative Tx: Yoga, Tx touch, meditation, poetry, journaling, acupuncture, art –Many saw CA survivorship as a “wake-up call” for change/reflection	
Fromme et al. 2004, **U.S.**^47^	Five MDs (22%) with BC ∼∼∼∼∼∼∼∼∼∼ ∼∼∼∼∼∼∼∼∼∼ Entire group: 23 MDs 10 female Current or previous CA Tx Mean age: 55 (28–83) Convenience sample of MDs identified by oncologists at the Authors’ institution **Semi-structured interviews**	Stage I BC Bilat Mx No further clinical details for the other four MDs with BC ∼∼∼∼∼∼∼∼∼∼ ∼∼∼∼∼∼∼∼∼∼ Entire group: Other CA -Five: prostate -Four: renal -Two: colon -Three lymphoma -One MD each: Bone, brain, larynx, thyroid, H & N -One MD had 2 CAs Illness stage: -Nine: >5y Dis-free -Five: >6 M Dis-free -Four: <6 M Dis-free -Two: In Tx -Three:MET/rapidly progressing CA	Small mass detected by Gynecologist who considered it a fibro-adenoma, but she suspected BC, arranged Dx & surgical consult herself ∼∼∼∼∼∼∼∼∼∼ ∼∼∼∼∼∼∼∼∼∼ Entire group: All but one opposed “self-doctoring”, but most cited instances when they did so (ordering Dx tests, Tx clearly CA-related symptoms) Recognize danger of denial	Med sub-specialist ∼∼∼∼∼∼∼∼∼∼ ∼∼∼∼∼∼∼∼∼∼ Entire group: Median y in medical practice: 19 (0–56) -Six: Pediatrics -Five: Family med /IM -Five: Neuro/Anes/ EM/RT Onc -Four: Adult sub-specialist -Three: Surgeon -11 University hospital -Nine: Private practice -Two: Community Hospital -One: Research/non-practicing	A question derived from the experience of one or more of the participants: “Are your nonmedical needs (rest, recreation, time off, decreased responsibilities at work) being met?” (p. 305) However, no explicit answer was given to this question.	∼∼∼∼∼∼∼∼∼∼ ∼∼∼∼∼∼∼∼∼∼ Entire group: RTW not explicitly described// Some implicit disclosure by ordering Dx tests for oneself	The noted question: “Are your nonmedical needs (rest, recreation, time off, decreased responsibilities at work) being met” (p. 305), although not directly answered, raised a further somewhat related, also unanswered question: whether their response was “more like a physician” or “more like a patient”?	∼∼∼∼∼∼∼∼∼∼ ∼∼∼∼∼∼∼∼∼∼ Entire group: MDs “have the same fears and difficulties as anyone else…need to give themselves permission to act like a normal patient.” (p.303), Need to relinquish control	The Authors consider that their findings suggest a need to strike a balance between the roles of patient and physician vis-à-vis cancer diagnosis, treatment and aftermath. Gender according to CA Dx not consistently stated
Goss et al, 2014, **UK**^28^	45 clinicians among 117 female HCW referred to occupational MDs post BC Dx. Age at 1^st^ referral to OH physician: <40: 13(11%) 40–60: 92(79%) >60: 12 (10%) **Retrospective case review**	All BC -82 (70%) CTx -11 (9%) documented distant MET	Not described	Not described	Five with LE	111 RTW, 98 RTW within 12 M/ Disclosure per se not explicitly reported	–Two: No modifications –97: Temporary adjustment of WH or duties, then RTW to original job –Six: Permanent adjustment of WH or duties (including three who had LE) –Six: Changed job One MD, one nurse “resumed full and usual duties after a gradual [RTW] programme” (p.636)	Not described	Relatively large study of health professionals with BC. Actual occupation is only described in certain limited cases. Therefore, generally cannot distinguish who were among the 45 clinicians.
Mitchell et al, 2015, **UK**^48^	∼∼∼∼∼∼∼∼∼∼ ∼∼∼∼∼∼∼∼∼∼ Entire group: Six nurses or midwives, seven allied health professionals (age 25–65) -All female All Tx for CA in last 2 y -Recruited via posters at work in local NHS setting in 3 counties + media **Semi-structured interviews**	Seven with BC (54%) ∼∼∼∼∼∼∼∼∼∼ ∼∼∼∼∼∼∼∼∼∼ Entire group: Other CA (of): -Two: Ovary -One: CRC -One: Thyroid -One: Brain -One: “Rare” type ≥ 6 M post Tx completion	-One participant noted nipple indent, delayed Dx ∝ denial ∼∼∼∼∼∼∼∼∼∼ ∼∼∼∼∼∼∼∼∼∼ Entire group: -Over-assumption re: knowledge by caregiver -One participant described initial detection from reading her blood test	∼∼∼∼∼∼∼∼∼∼ ∼∼∼∼∼∼∼∼∼∼ Entire group: Some responsibility for patients with CA, employed in NHS (full or part-time)	-Threat of job loss ∝ LE, need for long sleeves for symptom relief (to ↓ fluid) was against hospital policy ∼∼∼∼∼∼∼∼∼∼ ∼∼∼∼∼∼∼∼∼∼ Entire group: –Fatigue precluded night call –Some managers encouraged RTW –Cognitive performance hindered by CTx	∼∼∼∼∼∼∼∼∼∼ ∼∼∼∼∼∼∼∼∼∼ Entire group: 100% RTW// Most admitted to Onc center where they worked, sometimes in direct contact with own patients, some hid Dx, some disclosure discomfort, caution with patients	-One participant: Restricted arm stretch impact on hanging up a drip bag ∼∼∼∼∼∼∼∼∼∼ ∼∼∼∼∼∼∼∼∼∼ Entire group: Individual examples: -Gradual RTW with initial ↓ WH & # days /week -Works part-time, emergency night call problematic -Problem performing phlebotomy -Has become more “proactive” to help patients with CA in their decision-making -↑ difficulty of palliative care -One nurse felt obliged to retire	∼∼∼∼∼∼∼∼∼∼ ∼∼∼∼∼∼∼∼∼∼ Entire group: Individual examples: -Knowledge could → anxiety ∝ poor prognosis –Recall of patient deaths in room where she was receiving Tx	Health professionals noted to have had “distinctly unique experiences” ∝ being both patient & provider Few specifics for the participants with BC Each participant made personal decisions as to whether or not she would share her cancer experiences with her patients
Edward et al 2017, **Australia** ^29^	Six with BC ∼∼∼∼∼∼∼∼∼∼ ∼∼∼∼∼∼∼∼∼∼ Entire group: Eight female nurses Mean age: 57 ± 10.5 **Narrative**	-P2: Lx + sentinel node Bx. -P5: BC (no further specifics) -P8: Lx ∼∼∼∼∼∼∼∼∼∼ ∼∼∼∼∼∼∼∼∼∼ Rest of group: One with Hodgkin's lymphoma, One with melanoma 6 M to 20Y post-Tx.	-P2: Dx told by oncologist -P8: Called by radiologist re: abnormal MM ∼∼∼∼∼∼∼∼∼∼ ∼∼∼∼∼∼∼∼∼∼ Rest of group: One participant (P7) whose Dx is unstated wanted to be directly informed, even “over the phone” (p.1171)	∼∼∼∼∼∼∼∼∼∼ ∼∼∼∼∼∼∼∼∼∼ Entire group: Melbourne metropolitan hospital staff Years in nursing: 34.5 ± 5.5	∼∼∼∼∼∼∼∼∼∼ ∼∼∼∼∼∼∼∼∼∼ Entire group: –“Two-world knowledge as nurse & patient” helped RTW –Flexibility from supervisors//Some explicitly decided not to disclose, others chose Tx at workplace	-P2 & P8: //Strove for non-disclosure -P8: ↑ empathy with patients $\bar{p}$ own experience with CA. Supportive supervisor, flexible for time off for appointments ∼∼∼∼∼∼∼∼∼∼ ∼∼∼∼∼∼∼∼∼∼ Entire group: –Support from supervisors deemed important, especially time off for Tx. & to be able to continue working –With non-disclosure, in some cases apparently no workplace modifications		-P2: Antipathy at operation, elsewhere describes humor -P5: Avoids dwelling on things, emotionally stable -P8: focused on living, strove to be happy ∼∼∼∼∼∼∼∼∼∼ ∼∼∼∼∼∼∼∼∼∼ Entire group: -Importance of optimism/ Active coping to regulate emotions	The Authors emphasize the potential contribution of nurses who have survived cancer and RTW, as “practice role models”.
Lagad et al 2019 **Australia**^49^	∼∼∼∼∼∼∼∼∼∼ ∼∼∼∼∼∼∼∼∼∼ Entire group: 26 female HCW Age -40–49: 15% -50–59: 46% -60–69: 31% → 70: 8% **Semi-structured interviews**	-17 (65%) BC Explicitly noted: -Three: Stage 1 -Four: Stage 2 -Three: Stage 3 ∼∼∼∼∼∼∼∼∼∼ ∼∼∼∼∼∼∼∼∼∼ Entire group: -24 (92%) surgery -16 (62%) RT -Nine (35%) CTx -Nine (35%) HT -11: Stage 1 -11: Stage 2 -Four: Stage 3 Other CA: -Three: CRC -Two: Skin -One: Salivary gland -One Endometrial -One: Anal -One: Hematologic	- As a radiologist, she knew from the film: 95% 5-y survival Stage 1 BC ∼∼∼∼∼∼∼∼∼∼ ∼∼∼∼∼∼∼∼∼∼ Entire group: -Some: ↑ assertiveness to have concerns heard by providers -Some: awareness helped decision-making -↑ active participation in care ∝ professional background -Most chose the contrary: to be Tx as a “normal patient”	∼∼∼∼∼∼∼∼∼∼ ∼∼∼∼∼∼∼∼∼∼ Entire group: All with current or previous clinical experience in Oncology (46% > 20y) -Eight: Specialist/consultant Nurses -Seven: Nurses -Three: Physicians -Three: Social workers/counselors -Two: Clinical psychologists -One: Radiologist -One: Radiation therapist -One: Physiotherapist	∼∼∼∼∼∼∼∼∼∼ ∼∼∼∼∼∼∼∼∼∼ Entire group: 39% ↓ work capacity $\bar{p}$ Tx - Over half had emotional difficulties with patient contact, fear of recurrence	//Avoided use of support services ∝ privacy (Social worker Stage 2 BC ∼∼∼∼∼∼∼∼∼∼ ∼∼∼∼∼∼∼∼∼∼ Entire group: -42% worked during Tx -77% returned to original occupation// >50% viewed disclosure as “added component of practice” (p.5) but some chose not to disclose Special arrangements to maintain privacy when Tx at own workplace	Senior Nurse with Stage III BC -↑ “active involvement in patient care” -↑ empathy for patients based on experience Social worker with Stage II BC -Overly emotionally involved with very ill patients ∼∼∼∼∼∼∼∼∼∼ ∼∼∼∼∼∼∼∼∼∼ Entire group: Δ /impact on clinical practice: -Deeper communication with patients, especially Ψ aspects, ↑ “authentic” empathy -Some became “overly emotionally involved” disturbed by daily reminders of CA,…”overly invested in patients”, ↑ fear of recurrence (p.5) -Seeing patients with same Dx + MET: very difficult for a Nurse with melanoma	-Mainly viewed being informed as helpful, e.g., Radiologist with Stage 1 BC & Nurse with Stage II BC -Some ↑ fear (projected worst case scenarios), including MD with Stage I BC -Initial shock with Dx ∼∼∼∼∼∼∼∼∼∼ ∼∼∼∼∼∼∼∼∼∼ Entire group: -Those with counseling backgrounds proactively sought Ψ support	It is noteworthy that all the participants were health professionals working within oncology The Authors consider that guidelines are needed to address the cancer-related needs of health professionals with cancer
Jin & Lee, 2020, **South Korea** ^50,51^	∼∼∼∼∼∼∼∼∼∼ ∼∼∼∼∼∼∼∼∼∼ Entire group: 115–130 female nurses Mean age: 46.8 ± 8.6 **Cross sectional study**	66 BC (50.8% of participants) ∼∼∼∼∼∼∼∼∼∼ ∼∼∼∼∼∼∼∼∼∼ Entire group: CA Stage: 68.5% I 23.8% II 7.7% III Other CA: -39: Thyroid -11: Gastric -14: Other All “survivors”, with apparent definition: “had returned to work for six months after acute cancer treatment.” ^51^(p.3)	∼∼∼∼∼∼∼∼∼∼ ∼∼∼∼∼∼∼∼∼∼ Entire group: Not described	∼∼∼∼∼∼∼∼∼∼ ∼∼∼∼∼∼∼∼∼∼ Entire group: 63% Staff nurses 37% Nurse manager 62% > 21y nursing tenure Employed in general hospitals or clinics	∼∼∼∼∼∼∼∼ ∼∼∼∼∼∼∼∼ Entire group: ↑ Fatigue ∝ ↓ age (*)	∼∼∼∼∼∼∼∼ ∼∼∼∼∼∼∼∼ Entire group: 100% RTW (study selection criterion) -52% within 6 M, -76.9% to same workplace// CA disclosure reluctance noted among Korean nurses	66 nurses with BC: –Quality of Nursing Working Life (QNWL) Lowest of all CA types (**) –Expanded Nursing Stress Scale & fatigue: NS differences compared to other CA types ∼∼∼∼∼∼∼∼ ∼∼∼∼∼∼∼∼ Entire group: Working conditions described $\bar{p}$ RTW: –70% dayshifts, 30% 2 or 3 shifts –Harmony between workplace & the individual ∝ QNWL (***) –Nursing workplace spirituality ∝ QNWL (**) –↑ age ∝ ↑ QNWL (*)	∼∼∼∼∼∼∼∼∼∼ ∼∼∼∼∼∼∼∼∼∼ Entire group: ↑ Workplace spirituality ∝ ↑ age (***)	-The Authors highlight the importance of “harmony between workplace and individual” and “relationship with colleagues” in relation to quality of work life among nurse cancer survivors ^50^ (p.353) -Measures are needed to fortify bonds with colleagues & for multi-level attention to the needs of nurse cancer survivors, especially regarding their quality of work life. -Limited information about the nurses with BC -It is difficult to know what were the work conditions prior to the cancer diagnosis
Zavrtanik Čelan & Prosen 2022 **Slovenia**^52^	Five female nurses Age 41–51 at Dx, 48–52 at Interview **Semi-structured interviews** (2–10 y $\bar{\;p}$ Tx)	“Early” BC -Four: CTx -Two: Mx -Four: HT -Four: RT -One biological Tx -One axillary lymph node excision -One breast reconstruction -Physiotherapy + psychosocial support explicitly noted	One nurse described emotional reaction with learning the Dx from MD	Middle level nurses or medical technicians	-Fatigue most prominent issue -Cognitive deficits also of concern -Recommend “gradual” RTW, beginning with visits during illness –Support from colleagues & leadership helpful -UE problems ∝ radical axillary surgery -Noise distraction ∝ cognitive SE -Financial consideration & social contacts ∝ RTW	All yes, 13–39 M $\bar{\;p}$ Tx // Elastic glove explicit to patients, some patients expressed gladness that the nurse had RTW	–↓ WH for most (but could impair continuity) also →↓ salary & promotion prospects – COVID-19 pandemic→ particular concern ∝ impaired immune system. One nurse stated: if patient had positive COVID, she would stay home. But also reports of working on the COVID ward. –Colleagues covered for difficult tasks, but some complained about working extra -Note-taking described as a strategy to help complete tasks -Need for flexibility noted, especially re: scheduling & workload -Possibility offered to Δ to a telephone job, but no trial period offered	–Patient gladness re: RTW elicited good feelings -Family help & support vitally important	-This small study exclusively of nurse BC survivors illustrates the complexity of their RTW process -There is a particular need for coordination/ cooperation with supervisors & colleagues to promote support & understanding
You et al. 2024 **U.S.** ^53^	Eight nurses BC (47%) ∼∼∼∼∼∼∼∼∼∼ ∼∼∼∼∼∼∼∼∼∼ Entire group: 17 female nurses with CA Mean age: 51.8 ± 9.2 **Focus group study**: **Thematic analysis**	∼∼∼∼∼∼∼∼∼∼ ∼∼∼∼∼∼∼∼∼∼ Entire group: -Two: Gyn CA -Two: CRC One each with: -Hodgkin's -Leukemia -Melanoma -Thyroid CA -Anal CA Stage I – IV CA (65% Stage I or II) Surgery: 77% CTx: 82% RT:53% HT/immunoTx:24%	∼∼∼∼∼∼∼∼∼∼ ∼∼∼∼∼∼∼∼∼∼ Entire group: Not described	∼∼∼∼∼∼∼∼∼∼ ∼∼∼∼∼∼∼∼∼∼ Entire group: 65% Registered nurses 59% in ambulatory healthcare services Oncology nurses: ##9, 15 Some worked night shifts	∼∼∼∼∼∼∼∼∼∼ ∼∼∼∼∼∼∼∼∼∼ Entire group: RTW motivated by financial & intrinsic reasons (being “passionate” about nursing + supportive WE) Cognitive dysfunction, fatigue, neuropathy hindered RTW	∼∼∼∼∼∼∼∼∼∼ ∼∼∼∼∼∼∼∼∼∼ Entire group: 82% RTW during Tx//Discomfort with involuntary disclosure, some disclosed but others chose not to. Some selected care away from where they were employed	∼∼∼∼∼∼∼∼∼∼ ∼∼∼∼∼∼∼∼∼∼ Entire group: -A few continued without Δ, while Δ Employment status $\bar{p}$ Dx: 53% -Many Δ schedule, position, responsibilities -Took time off (paid &/or unpaid) during CA Tx: 53% -To handle cognitive challenges: arrived early to review details about patients -Chose work units where they felt more comfortable -Reduced assignment to isolation unit explicitly noted by one nurse during COVID pandemic –Experience with CA enhanced insights for their role as nurses & for patient care but also could trigger emotions –Nurses in administrative roles: ↑ empathy for staff nurses with CA –One nurse described accommodating supervisor who understood that working quickly was difficult –Nurse #6: requested dayshift, had worked nights	∼∼∼∼∼∼∼∼∼∼ ∼∼∼∼∼∼∼∼∼∼ Entire group: –Support from family & friends helped ↓ distress	-This study provides many rich insights into RTW among nurses with cancer, of likely relevance for BC. However, no further specific information about these eight nurses with BC who comprised nearly half of the participants.
Mao et al. 2025 **China** ^54^	-11 Nurses -Nine MDs All female Participants selected to have no mental illness Mean age: 38.5 **Semi-structured interviews**	All primary (not recurrent) BC with modified radical Mx Not terminal stage	Not described	13–20 y of healthcare experiences in various medical fields (oncology per se not mentioned)	-All desired RTW -Most continued work during CTx -Guilt feelings during sick leave noted -Supervisors may not approve extending sick leave -A capable assistant facilitated an MD's earlier RTW -Fear of recurrence ∝ RTW -Worry re: COVID & recurrence -↓ memory, energy -Intolerance to crowded spaces -An MD worried that fatigue could → errors -RTW motivated by “being needed” for competence, financial advantage (MDs)	–All RTW 28–112 D sickleave// Disclosure per se not mentioned	-Δ duties: ↓ workload, eliminated night shift -↑ sensitivity to patients with CA -Nurse #11: even with ↓ nightshift, physical capability surpassed, needed further WE Δ's -Nurse #15: ↓ surgery, found teaching students fulfilling	-Anxiety re: recurrence -Several MDs devoted ↑ time to family $\bar{p}$ BC	-Numerous helpful insights re: RTW exclusively among physicians & nurses with BC. -Specifics re: CTx and other post-surgical Tx not described.

Statistical significance * p < 0.05, ** p < 0.01*** p < 0.001.

Anes = Anesthesia, BC = breast cancer, Bilat = bilateral, CA = cancer, CRC = colorectal cancer, CTx = chemotherapy, Dis = disease, Dx = diagnosis, diagnosed, EM = emergency medicine, Gyn = gynecologic, HCW = health care workers, H & N = head & neck, HT = hormonal therapy, ICU = intensive care unit, IM = internal medicine, LE = lymphedema, Lx = lumpectomy, M = month, MD = physician, Med = Medicine/medical, MET = metastatic disease, MM = mammogram, Mx = mastectomy, Neuro = Neurology, NHS = National health service (UK), NS = statistically non-significant, OH = occupational health, Onc = oncology, P = participant, QNWL = quality of nursing work life, RT = radiation therapy, RTW = return to work, SE = side effect, Tx = treatment/treating, UE = upper extremity, UK = United Kingdom, U.S. = United States of America, WE = work environment, WH = work hours, y = year, Δ = change, Ψ = psychological, $\bar{p}=$ post (after), ∝ = in relation to.

**Table 3.** Case reports & self-reports of health professionals with breast cancer.

1^st^ Author, pub y, country	Profession, Age at Dx, Report type	Initial detection/ how Dx conveyed	Clinical status	Baseline job conditions	Issues directly relevant to RTW	RTW/ Disclosure	Work stressor status/ modification with RTW	Ψ/ lifestyle issues	Further comments
Kaelin 2005, **U.S.** ^55,56^	Oncologic breast surgeon Age: 42 **Self-report**	Self-detected slight skin indentation on right breast, normal initial MM but “dim shape” on ultrasound, needle Bx/Chief of breast pathology came to [her] office holding pathology slides …to gently and regretfully break the news [of BC]” (p.2) ^56^	5 surgeries: 3 Lx, Mx, breast reconstruction, multiple lesions, CTx, HT	Surgical oncologist, Director of Comprehensive Breast Health Center, –Prior to Tx. slept ∼6 h/night, recalls “many sleepless nights” during 5y surgical residency, being “well used to working through fatigue” (P.238)	–Neuropathy –Extreme fatigue –Importance of social support from colleagues, staff & patients	Modified RTW, timing not specifically noted /Explicit disclosure via ^55,56^ further described therein	–Surgical practice precluded ∝ Neuropathy –Δ focus to BC education –Need to get “a good night's sleep every night…going to bed earlier and sleeping later than usual for a while” ^56^ (p.241) –Recommends: ↓ need for multi-tasking, Avoid “being plunged into emergency situations that call for a quick reaction” (p.241) –Importance of vacation noted	–When moving back “into the center stream of [one's] life, be gentle with (one's] self” ^56^ (p.279) –Importance of vigorous recreational PA	-Having written the book ^56^, it can be surmised to have been a way for her to find meaning & purpose through modified work, i.e., BC education, with outreach to a broad readership, offering “nitty-gritty guidance from [her] experience as a breast cancer surgeon who has also been a patient” (p.12) -In the book, it is sometimes difficult to know how much is actually her experience vs. advice to the reader
Consalvo, Piscitelli, et al 2007, **U.S.**^57^	Oncology nurse, Linda Piscitelli, including self-disclosure, report by supervisor (Consalvo), attending MD & employer (Mintzer) & staff (All authors of this paper) Age: unspecified **Case study**	Annual screening MM/Received a “frantic call from 1° care MD indicating a ‘questionable finding’”, BC Dx conveyed by radiologist who stated that she suspected BC & disclosed that she herself had had BC, with CTx & reconstruction, after which one can be “just fine” (p.229)	Surgery, open wound on chest, CTx, breast reconstruction	−8 y as an oncology nurse –“Large group of hematologists & oncologists”: POHA –“new employee: no benefits, no sick time, & no vacation time” (p.229)	–All Tx at POHA: her workplace –Neuropathy	–Decided to work during CTx to keep herself occupied/ Disclosure noted to be an inspiration to some patients, while others might not be comfortable receiving care from a nurse who is ill	−∝ time-consuming chest wound packing: Supervisor Δ WH from 8:30–17:00 to 10:00–16:00, which was deemed very helpful –Severe neuropathy→sick leave recommended by supervisor, who asked herself whether these measures exceeded her professional obligations	–Music & relaxation Tx identified as most helpful	–Extensive consideration of advantages & disadvantages of being Tx at one's own health center: Especially re: disclosure & also if complications arise -Through this case presentation, including self-report, a deep appreciation of the survivorship process is gleaned -“The courage of one individual's journey has demonstrated how a negative situation can be transformed into a positive one” (p.227)
Bush 2009 **U.S.** ^58^	Oncology nurse, Academic- Assistant Clinical Professor Age: Unspecified **Self-report** within a broader article on PTSD ∝ CA	Dx work-up during appendectomy/ How findings were conveyed to her not described	Stage I BC + Stage II Ovarian CA Surgery, CTx, RT, HT PTSD (described 5 M $\bar{p}$ CA Tx) PMHx: Infertility, in vitro fertilization×8, melanoma, anxiety/depression FHx: Sister BC, Son clinical depression, several other major illnesses	Oncologic Nurse Practitioner with clinical psychology as a specialty, Assistant Clinical Professor working in academic institution	-Disturbed sleep/CA-related nightmares -Fatigue -Cognitive dysfunction -Peripheral neuropathy -Advice from radiation oncologist re: RTW timing: 8 wk $\bar{p}$ RT	To academic work sometime w/in 1y/ Avoided encounters with known persons (Not explicitly at work)	Resigned “rewarding job I cherished” as a nurse practitioner: Could not “physically, emotionally or cognitively do the work in fairness to myself or my patients” (p.396) States: “multitasking, time organization…endless intellectual input & politics of an academic environment can often be overwhelming” (p. 399)	-“Grieving the loss of…self-image” (p.397) -Practices self-care -Strives to “hold onto those aspects that have served [her] well…kindness, compassion, optimism, humor, love of others… [&] let go of [those] that may have … drain[ed] emotional reserve.” (p.399)	-Illustrates the powerful compounding of BC + other malignances + PTSD + family history, as these deleteriously impact on RTW into clinical work within oncology -Written both as a clinical article on PTSD & CA & relevance for oncology nurses, & with inserts about herself
McCorkle 2012 **U.S.**^59^	Oncology nurse, PhD Age: Unspecified **Self-report**	“No warning signs or symptoms” (p.245), no further description/ How findings were conveyed to her not described	Surgery, CTx, RT Self-described PTSD	Not explicitly described Notes past work experience as a flight nurse in a combat region: clinical experience with PTSD	-LE -Fatigue	Gradual to full time/Disclosure not discussed	-Colleagues covered much of her Admin tasks -With RTW to clinical duties found listening to patients describing their problems as overwhelming -“Being a patient [gave] insights to the personal courage that it takes to endure the despair” (pp.245–6) -Finds helping patients “get through their cancer experience…amazingly gratifying” (p.246)	“Particularly difficult for nurses to ask for & accept help” (p. 245)	The paper was published 22 y after her Dx. This longer range perspective illustrates the potential of this oncology nurse to transform her own BC experience into ↑ capacity to help patients This experience has been particularly valuable in helping patients “get through their own CA experience the best they can” (p.246)
Belkić, Savić, 2013, Country unspecified ^60^	Oncology nurse Age: late 50s **Interventional** **case study**	Routine biennial MM (non-palpable microcalcified lesion) followed by excisional Bx/ How findings were conveyed to her not described	Stage I BC, Lx, RT, HT PTSD Nulliparous BMI = 27.8	–30 y as an oncology nurse Healthy social climate, but low pay, 2^nd^ job, night shift, long WH, EM/ICU work, patients with endstage CA, Admin, very high job demands, some ERI, high TAV, some conflict, heavy physical lifting Total OSI=85	-Disturbed sleep, -Cancer-related nightmares -Heavy coffee intake	Yes/ Selective disclosure	–Negotiates ↑ pay, quits 2^nd^ job, ↓WH –Day shift only –Avoids work with patients with endstage CA (as recommended by the occupational neuropsychiatrist) –↓ Admin, delegate work to other staff (Heavy lifting was apparently not modified with RTW) Total OSI=72.75 (↓ 12.25)	Referral to occupational neuropsychiatrist deemed helpful Via CBT: Expresses CA-related fear, ↓ over-commitment ↑ recreational PA (dance) ↓ coffee intake	-Illustrates the powerful compounding of BC + PTSD, with appropriately modified work conditions + attention to ψ/lifestyle issues → healthy RTW -“Competence & dedication highly appreciated by patients, colleagues, staff, & leadership within the hospital… articulates satisfaction she feels from her work… lifelong commitment to fight against the scourge of cancer acquire[s] a new dimension of poignancy and urgency”(pp.214–5) -The BC was earlier stage than for most of the other cases
Silver 2013, 2015 **U.S.**^61,62^	MD Physical Medicine/ Rehabilitation Specialist, Academic-Associate Professor Age: 38 y **Self-report**	2 y prior to Dx self-detected thickness in breast (age 36), MM + ultrasound normal×2 (she had pursued a 2^nd^ opinion), Notes: negative FHx & at that time, she was considered “too young to have a baseline MM on file” (p.31) Age 38: F/u of the “same spot” [indicated] the BC “was obvious” (p. 31) ^62^	-Surgery (no further details) -CTx Dose-dense regimen -Stage not explicitly described, only that “prognosis was better than [she] initially suspected” (pp.178–9) ^62^	Physician & founding Medical Director of Outpatient Rehabilitation Hospital “busy doctor struggling to balance work and family” (p.4) ^62^	–Clinical/scientific expert in RTW –Recommends: “Pre-habilitation…specific assessments and interventions…to help [those] newly diagnosed…to prepare for upcoming Tx” (pp. 6–7) (but unclear whether she herself did this vis-à-vis RTW) –Extreme fatigue described during CTx, 1y later in the afternoons, –Painful peripheral neuropathy & was “hunched over” (p.109) –Expresses concern about her appearance –Poor physical endurance –Disturbed sleep –“want[ed] to take care of [her] patients” during CTx, yet realized that she “couldn’t manage to do most of what [she] usually did” (p.217) –Advised by oncologist to RTW “when [she] felt well enough” (p. 473) ^62^	-Resumed role as MD $\bar{p}$ completing Tx//Implicit disclosure to patient(s) during Tx -Describes request by patient's wife to talk to the patient who “saw what [she] went through and believes [she could] help him” (p.242) ^62^	–Developed the STAR (Survivorship Training and Rehabilitation) program –Began plan to write the book ^62^ 4D $\bar{p}$ RTW –Luncheon in honor of several including Dr Silver 1 h drive to work: notably difficult –Very little about her actual work conditions with RTW	-Recommends redirecting fear to motivate measures towards healing (p.13) -Addresses telling family (children) what one can and cannot do (p. 229) -Strongly advocates exercise, she herself strove to walk vigorously & do elliptical training during & immediately $\bar{p}$ CTx (p. 107) ^62^	-The Author has “developed a new standard for [cancer] rehabilitation” through her own experience (p.474) ^61^ -In the book, it is not always clear how much is actually her experience vs. advice to the reader
Mapi, 2018 **Philippines**^63^	Nurse, Master's degree Age: 43 **Self-report**	Self-detected a “tiny lump” (considered initially pre-menstrual), which grew over a few M, pain prompted her to seek medical attention/Initial Dx (BI-RADS V) conveyed in a sealed envelope	Invasive ductal BC stage 2A, modified radical Mx, CTx	At the time of BC Dx: “considered as the peak of [her] career as a nurse, when [she] felt [she] had all the skills, knowledge, and confidence to deliver at [her] very best the nursing care needed by [her] patients” (p.291) & was pursuing a Master's Degree in Nursing	–Support from family & friends implied as helping RTW	Took leave from work & academic pursuit, RTW $\bar{p}$ CTx & short vacation/ Disclosure implied, but not specifically addressed	–Full time nursing work + graduate work: completed her Master's Degree focusing on Nurses with cancer –Very little about her actual work conditions with RTW	–Describes difficulty in disclosing to her children –Drastic Δ in appearance⇒”feeling the pain, misery, and the truth of being a nurse afflicted with CA” (p.294) –Importance of spiritual dimension/resiliency	-Poignant description of the experience of BC by a nurse -Her own experience motivated interest in the CA experience of other nurses
Sun & Armer, 2019, **U.S.**^30^	Nurse Counseling Master Age: 38 at Dx, 65 at report **Case study**	-Initial detection of BC & how findings were conveyed to her not described -Difficulties in the clinical Dx & Tx of LE described	Initial Lx, 20 nodes excised, RT, recur, Bilat Mx, Hx, LE: 3y $\bar{p}$ BC Dx –Comorbidities: anxiety, depression, hypothyroidism	**School nurse**: 70–100 children/day comprehensive screening, clinical care of pupils who were ill, immunization, education to pupils, parents & staff, home visits, 40 h/wk	2 M sick leave with Tx, time off for clinic visits, employer medical coverage	-Worked full time -Several job Δ's / Disclosure not explicitly addressed but most likely yes ∝ LE	–↓ heavy lifting/carrying (problematic since part of work): used roller bags –“Bandages/compression sleeves distracted from job role” (p.24), –↑ infection risk—quick response: But a major risk when working as school nurse & with work-related travel -Among job Δ's: coordinator of CA, which required travel –difficult for swollen arm -Use of computer as a major form of work	-Shares coping strategies -Lack of LE Dx → frustration & anger -Emotional distress ∝ bandaging -Fear ∝ infection risk	-Describes the multifactorial complexity of her RTW experience vis-à-vis BC-related LE as a nurse -Notes that “with each new challenge, (she] was able to evaluate barriers and consider material and strategic resources for coping” (p.26)
Thompson Buum, 2019 **U.S.**^64,65^	1° care MD/ internal medicine Age: 44 **Self-report**	Lump felt on self-exam (had not yet begun MM screening)/ Notified that Bx revealed BC	Surgery, HT, post-operative axillary web syndrome No recur at 1y f/u	>15 y work in 1° care, with a substantial number of patients with BC	Received BC care at workplace	RTW apparently quite rapid though not explicitly described /Implicit disclosure to colleagues, selective disclosure to patients	–↑ number of patients with BC –With appropriate precautions, email communication with patients appears to be useful & not overwhelming –Open lobby waiting room as a patient—impinges on privacy but also source of candid, mutually supportive interactions –Describes a patient whose wife has MET, she is “reminded of the complexity of breaking bad news” – Queries about how to convey the “bad news” to children—the very issue this physician has described in her own family: and writes the present essay ^65^	Acknowledges her own fear & sadness, as well as optimism, in conjunction with disclosure to her children/ Music, writing & exercise as coping strategies/ celebrated 1 y CA-free f/u	-Raises questions regarding disclosure to patients as sometimes “too much information” while some “patients appreciate the ability to share and connect, including knowing that their doctor has been through something similar” (p.173) ^64^ -Enjoys “newfound synergy and expertise” relating to various CA-related issues vis-à-vis patients (p.174) ^64^ –Addresses many issues about the emotionally-disturbing aspects of CA, appears to have found helpful, effective strategies

Admin = administrative, BC = breast cancer, Bilat = bilateral, BMI = body mass index, Bx = biopsy, CA = cancer, CBT = Cognitive/behavioral therapy, CTx = chemotherapy, Dx = diagnosis, EM = emergency medicine, ERI = effort-reward imbalance, exam = examination, FHx = family history, f/u = follow-up, HT = hormonal therapy, Hx = hysterectomy, ICU = intensive care unit, LE = lymphedema, Lx = lumpectomy, MD = physician, MET = metastatic disease, MM = mammography, Mx = mastectomy, OSI = Occupational Stressor Index, PA = physical activity, PMHx = past medical history, POHA = Pennsylvania Oncology Hematology Associates, PTSD = post-traumatic stress disorder, recur = recurrence, RT = radiation therapy, RTW = return to work, STAR = Survivorship Training and Rehabilitation, TAV = threat avoidant vigilance, Tx = treatment/treating, U.S. = United States of America, WH = work hours, w/in = within, wk = week(s), y = year(s), Δ = change, Ψ = psychological, $\bar{p}=$ post (after), ∝ = in relation to.

### Studies of RTW among women with breast cancer, of various occupational profiles, including health professionals

Table 1 presents the 14 separate studies of RTW among women with BC, in which some of the participants were health professionals.^16,^^22–26^^,37–45^ Most of these studies were various types of small-scale narrative interviews, in which nurses, physiotherapists, physicians and/or health professionals of other (or undefined) profiles were included. Oncologic treatment of the interviewed health professionals was varied, but, in addition to surgery, many received chemotherapy. Relatively little was reported regarding their baseline working conditions, except occasional mention of work hours (often long), nightshift work and busy schedules/need for multi-tasking.^23,24,38,41,42^ Particular concerns for health professionals vis-à-vis RTW that arose regarding chemotherapy included infection risk,^37^ fatigue,^41,43^ impact upon cognitive function^24,38^ and upon appearance.^25,42^ The latter was a sensitive issue often linked to disclosure of BC, especially to patients.^25^ Shorter work hours, nightshift work and/or taking proper breaks for meals became explicit issues with RTW for some of the nurses^23,24,39,41^ and one of the physicians.^26^ The emotional burden of dealing with patients’ health problems while being afflicted with BC was also poignantly expressed.^37^

Some salutogenic developments could also be identified among the health professionals with RTW. These included asking colleagues for help e.g., with challenging clinical procedures.^25^ One nurse stated that she had learned a great deal by being a patient. With RTW, she was better able to set boundaries, avoiding over-involvement outside work hours, i.e., reducing intrinsic ERI.^23^ One physiotherapist considered gradually implemented RTW as having helped normalize her life.^22^ Presumably BC had been detected at an early stage for this physiotherapist, since besides surgery, she received only hormonal therapy. However, further clinical details were not described in her case. Both physiotherapists participating in^22^ resumed work at 12 months. Satisfaction associated with their specific job profile may have helped promote RTW.

For all the participants included in these studies, a key finding regarding RTW was the importance of work-place social support. Both instrumental and emotional support from supervisors and co-workers were repeatedly cited. Concordant with that support was the implementation of various workplace modifications. The most frequent of these were changes in job tasks and work hours/schedule. Of particular importance, often in direct relation to the latter, was the allocation of needed time for treatment/medical appointments. Some specific ergonomic modifications were described,^26,44^ especially in relation to special needs. Among these were work station adaptations, reportedly associated with high satisfaction for women with upper limb lymphedema.^44^

Among 73 women whose BC treatment included axillary lymph node dissection and mastectomy, RTW at three months was reported to be nearly 50%. However, the percentage was notably lower among the “care workers” (36%).^45^ The impact of upper limb lymphedema on work-related activity was substantial for many health professionals, especially if the dominant arm was involved.^44^

### Studies of health professionals with breast cancer and other malignancies

Ten separate studies were identified addressing RTW among a series of health professionals with cancer and in which BC was the diagnosis for at least some of the participants ^27–29^^,46–54^ (Table 2). Of these, BC was the sole diagnosis in three publications.^28,52,54^ In the following studies: ^27,29,46^^50,[Bibr bibr51-10519815251410109][Bibr bibr52-10519815251410109]–53^ only nurses were included, whereas only physicians were included in reference.^47^ Nurses and physicians were participants in,^54^ whereas various categories of health professionals were listed in.^28,48,49^ Most of the studies were of female health professionals exclusively, whereas the majority of the participants were male physicians in.^47^ Two male nurses with malignancies other than BC were included in.^27,46^

Altogether, 264 health professionals with BC were participants in the studies summarized in Table 2. Limited oncologic detail can be gleaned about them. Lumpectomy was the cited surgical treatment for two nurses,^29^ while ten physicians and 14 nurses had undergone mastectomy.^47,52,54^ Explicitly, four health professionals had Stage 1 BC, four had Stage 2 and three had Stage 3 BC.^47,49^ Distant metastases (Stage 4) were documented among 11 of the 117 health professionals with BC included in.^28^ Chemotherapy was received by 82 (70%) of the participants in^28^ and four of the five nurses with BC in.^52^

The initial BC diagnosis can be particularly sensitive for health professionals. This issue and related topics are addressed in column 4 of Table 2. Three nurses were directly informed by a physician,^29,52^ with an emotional reaction described in one case.^52^ Another health professional self-detected nipple indentation, but due to denial, delayed the actual BC diagnosis.^48^ On the other hand, a medical sub-specialist who was told by her colleague that a small breast mass was benign, pursued the subsequent diagnostic workup herself. She underwent bilateral mastectomy for Stage I BC.^47^ A radiologist reportedly examined the film herself, noting that the Stage I BC was associated with a very favorable prognosis.^49^

Small-scale narrative interviews predominated for the studies included in Table 2, as was the case for those in Table 1. Except for some mention of job title, length of employment and setting, compared to the studies presented in Table 1, even less was reported about the baseline working conditions of the health professionals with BC or other malignancies. Only one study^53^ referred to nightshift work at baseline, noting that this was the case for some of the 17 nurses with various malignancies. Nearly 100% RTW, sometimes gradual, was reported in the three studies^28,52,54^ in which only health professionals with BC were included. In the two of those publications based on qualitative analysis,^52,54^ fatigue and cognitive deficits were major concerns, while social support as well as financial considerations reportedly promoted RTW.

Some changes in work conditions with RTW were reported in,^28,^^52–54^ most frequently shortened workhours, elimination of night work and change in duties, with actual change in jobs occasionally reported. While colleagues covered for difficult tasks, they sometimes complained about the extra burden.^52^ In a larger study from South Korea, the overall quality of nursing working life after RTW was reportedly the lowest among nurses with BC compared to nurses with other malignancies.^50,51^ Concern about workplace exposure to COVID-19 was noted in the most recent studies.^52–54^

Upper extremity problems were cited in,^28,48,52^ with the need to wear an elastic glove, necessitating BC disclosure to patients.^52^ A health professional with BC and lymphedema in the UK study^48^ reportedly faced job loss since long sleeves were not permitted, while restricted arm stretch impacted her ability to place a drip bag for intravenous treatment.

Among the emotional rewards of RTW for health professionals with BC were the feeling of being needed and enhanced sensitivity/empathy for patients with cancer.^29,49,54^ A patient's explicit expression of gladness when a nurse with BC returned was particularly poignant.^52^ Deeper insights and appreciation of the potential contribution to helping patients with cancer was likewise reflected among the health professionals with various malignancies.^27,46,48,49,53^ However, further work with patients afflicted with cancer, particularly end-stage disease, could also trigger adverse emotional responses.^48,49,53^ Disclosure of a cancer diagnosis to patients, coworkers and supervisors was a very personal decision, as well as being impacted by cultural considerations, with some health professionals opting to refrain.^27,29,46,^^48–51^^,53^ In some cases, the latter precluded implementation of needed workplace modifications.^29^

### Case studies/self-reports of health professionals with breast cancer

Nine published case studies/self-reports of female health professionals with BC were found.^30,55–65^ Among these, seven health professionals worked within some aspect of clinical oncology,^55–62^^,64,65^ including surgery,^55,56^ rehabilitation^61,62^ and primary care of patients with cancer ^64,65^ (Table 3). Six of these reports were from nurses,^30,^^57–60^^, 63^ while three were from physicians.^55,56,61,62,64,65^

The three physicians^55,56,61,62,64,65^ and one of the nurses^63^ initially self-detected a breast change, whereas BC was found during screening mammography for two oncology nurses.^57,60^ While the initial diagnosis of BI-RADS V (Breast imaging-reporting and data system) was conveyed in a sealed envelope for one of the nurses,^63^ personal communication was reported for the BC surgeon^56^ and for one of the oncology nurses.^57^ In the latter case,^57^ a radiologist who, herself, had undergone extensive treatment for BC provided explicit encouragement that after treatment one can be “just fine” (p.229).

Although the full clinical details were not usually reported, all but two of the cases involved health professionals with BC who had undergone mastectomy and/or chemotherapy.^60,64,65^ Upper extremity complications were described in three cases: axillary web syndrome^64^ and upper arm lymphedema.^30,59^ In addition to BC, one of the oncology nurses was also treated for ovarian cancer.^58^ She, as well as two of the other oncology nurses,^59,60^ reportedly suffered Post-traumatic stress disorder (PTSD) in association with the BC diagnosis and treatment. The physician rehabilitation specialist reported disturbed sleep, but not full-blown PTSD.^62^

Overall, there is rather limited information about the baseline job conditions in most of these case/self-reports. Exposure to nightshift work is explicitly noted by the oncologic breast surgeon^56^ and for one of the oncology nurses.^60^ Another oncology nurse^57^ as a new employee lacked benefits, including sickleave and vacation time. Besides clinical work, four of the health professionals also had academic and/or leadership responsibilities.^55,56,58,^^61–63^ A detailed assessment of baseline job stressors was provided for one of the oncology nurses.^60^ Notwithstanding a healthy social climate at the cancer hospital where she had worked for 30 years, high demands, lifting patients, heavy threat avoidant vigilance burden, as well as some conflict and effort-reward imbalance, ERI, contributed to a total Occupational Stressor Index, OSI score of 85. The latter is nearly at the level for which urgent intervention is needed, and, as mentioned, is associated with poor sleep quality.^9^

All of the health professionals returned to work in some capacity, mainly after treatment,^58,59,^^61–63^ although one oncology nurse chose to work during chemotherapy in order to keep herself occupied.^57^ Extreme fatigue and/or neuropathy were reported by nearly all the health professionals who had undergone chemotherapy,^55–59^^,61,62^ while cognitive dysfunction was also reported by the oncology nurse who had incurred ovarian cancer as well as PTSD.^58^

The oncologic breast surgeon,^55,56^ the rehabilitation physician^61,62^ and one of the oncology nurses^57^ described some favorable aspects of disclosure to colleagues, staff and patients. This included social support.^55,56^ However, the need to be selective was also emphasized.^57,60,64,65^

For several of the health professionals, major work modifications were made in association with RTW. Due to chemotherapy-induced neuropathy, the oncologic breast surgeon was obliged to refocus her professional efforts towards BC education.^55,56^ An oncology nurse who had endured PTSD received help to diminish administrative tasks. Notwithstanding the gratification of helping patients with cancer, she found that listening to patients’ problems could also be overwhelming.^59^ The oncology nurse who had suffered ovarian cancer as well as PTSD in addition to BC, resigned from clinical practice, turning instead to academic pursuits, although those were frequently overwhelming for her.^58^ In contrast, another nurse not only returned full-time to clinical practice, but also pursued a Master's degree focusing on nurses with cancer.^63^ After RTW, the primary care physician increased the number of patients with BC in her clinical practice.^64,65^ Several workplace modifications were made for the third oncology nurse who had suffered PTSD, lowering the total OSI score by 12.3 points. Among these modifications were a negotiated pay increase, allowing her to quit her 2^nd^ job, thereby reducing workhours, elimination of nightshift work and diminishing administrative tasks. In addition, the occupational neuropsychiatrist recommended that she avoid working with patients suffering end-stage disease. These work-related interventions, together with cognitive behavioral therapy, reducing job over-commitment and dance as recreational physical activity plus healthy social interaction, were associated with an overall favorable outcome. She thereby garnered renewed élan towards her “lifelong commitment to fight against the scourge of cancer”^60^ (pp. 214–5). The importance of attention to psychological/lifestyle issues is underscored for all the case reports of health professionals presented in this section.

## Discussion

### Integrative review—synthesis of the findings

In contrast to the plethora of studies on RTW among women with BC, relatively few explicitly included health professionals with BC. Regarding the latter, based on the present searches, the largest number of RTW publications were those in which some of the participants with BC were health professionals. However, this was often with little or no further information about the latter. For the 14 identified studies carried out among various occupational profiles, altogether there were at least 122 health professionals with BC about whom some work-related information could be gleaned. Regarding the 10 studies exclusively focused on health professionals, 264 participants had BC. These were among a substantially larger group of health professionals who had been afflicted with malignancies other than BC. Both males and females were included in some publications. In addition, nine case studies/self-reports of health professionals with BC were identified. For the publications devoted explicitly to health professionals with BC or other cancers, insights were limited regarding their work conditions prior to, during and after BC treatment.

Notwithstanding the lack of complete clinical data, it can be concluded that besides surgical intervention, a sizable percentage of the health professionals with BC included in this review had received chemotherapy. Major issues that arose in relation to chemotherapy included extreme fatigue, neuropathy, infection risk, cognitive dysfunction and impact on appearance. The latter was particularly delicate vis-à-vis disclosure to patients and to others in the healthcare work environment. With RTW numerous shorter and longer-term modifications were made due to chemotherapy. Perhaps the most dramatic was the need to completely redirect clinical practice from surgery to medical education because of chemotherapy-induced neuropathy.^55,56^ Physiotherapy and other non-pharmacologic strategies are being investigated to counter chemotherapy-induced peripheral neuropathy ^66,67^. If successful, such measures could help preserve the work capacity of affected health professionals, especially nurses, surgeons and other physicians who perform invasive procedures.

Lymphedema and/or other upper arm problems were also an issue specifically for the health professionals with BC in many of the studies.^23,28,30,44,45,48,52,59,64,65^ On the one hand, work-station modifications were reported to be helpful for employed women with BC-related upper arm lymphedema.^26,44^ On the other hand, lack of appreciation of the special RTW needs of nurses associated with this BC-related complication was also reported.^30,48^ A particular challenge was related to the increasingly-applied “bare below the elbow” (BBE) policy, aimed at reducing nosocomial infections.^68^ Efforts are ongoing to find solutions for health professionals who for cultural-religious reasons are conflicted by BBE policy.^69^ Such solutions could be helpful, as well, for health professionals with upper arm lymphedema. At the same time, strides made to optimize axillary management hold promise to minimize upper arm lymphedema as well as BC recurrence.^70^

The 24-year “journey” traversed by a nurse with BC-related lymphedema provides further insights into this complex problem.^30^ Her working conditions at baseline had been intense. As a school nurse and counselor, she was responsible for up to 100 children per day, with many additional duties. Apparently, she continued to work in that capacity for some time after the occurrence of lymphedema, although several subsequent job changes were also described.^30^

With a few other exceptions, very limited information is available concerning the baseline working conditions of the health professionals with BC. This hinders adequate assessment of RTW, including considerations of job stressors that may have contributed not only to the increased BC risk at that time, but that may also impact risk of BC recurrence. A particular weakness was the lack of attention to nightshift work. Altogether only seven of the 33 separate studies included in this review addressed diminution or avoidance of nightshift work with RTW in some way.^24,^^50–54^^,56,60^

As noted, a link between nightshift work and sleep deficiency has been demonstrated among nurses.^7^ The prevalence of severe insomnia, as well as overall PTSD symptoms, was substantial among health professionals working nightshifts in a study carried out in public hospitals during the COVID-19 pandemic.^71^ The relation between PTSD and the cancer experience is well-recognized.^58^ These considerations warrant attention for health professionals with BC, who may well be vulnerable to PTSD, and for whom attention to nightshift work could be an important component of interventions aimed specifically at improved sleep with RTW.

Another stressor, particularly salient for clinical work within oncology, is the emotional challenge of providing care for patients with advanced and/or endstage disease.^72^ Distress in this regard was articulated by health professionals in some of the studies included in this review.^48,49,60^ For one of the oncology nurses,^60^ given the presence of PTSD, including recurrent nightmares about patients with metastatic and terminal outcomes, the occupational neuropsychiatrist recommended that she avoid work with patients suffering endstage malignancy. Such an intervention could be an option, at least temporarily, in selected cases.

Post-traumatic stress disorder was explicitly diagnosed only in three of the case studies included in this review.^58–60^ More attention to the possibility of PTSD is warranted among health professionals with BC or other malignancies who provide care within oncology. Their emotions can be triggered as they over-empathize with patients who have similar cancer experiences.

On the other hand, continued clinical work, including within cancer care, was often a source of gratification. Many of the health professionals gleaned new-found insights from their own BC experience, with deepened empathy which could be of benefit to their patients.^23,27,29,46,49,^^53–57^^,59–65^ This was also poignantly illustrated by the manner in which the radiologist who herself had undergone major BC treatment, conveyed the initial BC diagnosis to an oncology nurse.^57^

In sharp contrast, was the nurse who described the “total shock” of receiving a diagnosis of BI-RADS V in “a sealed white envelope”^63^ (pp. 291–292). A comparably devastating manner of receiving a diagnosis of a highly malignant tumor is described by Dr Karla Castro-Frenzel, anesthesiologist, who was informed by a surgeon via “a gut-wrenching Monday-morning phone call at work after anesthetizing [her] first patient”^73^ (p. 2303). Conveying a diagnosis of BC or other malignancy to any patient is obviously exceedingly delicate, requiring the utmost sensitivity and skill.^74^ At the very least, such sensitivity and skill should be applied when the recipient of the “bad news” is a health professional. It could, in fact, be that health professionals have special needs in this regard. If these are not taken into account, untoward consequences may result. Among these could even be avoidance of BC screening. Of the limited published data on this topic, it appears that health professionals are underscreened for BC.^75,76^ These considerations become highly salient regarding adherence to needed follow-up. As noted, fear of recurrence was expressed by a number of the health professionals with BC,^49,54^ particularly those working within oncology.

Dr Castro-Frenzel eloquently describes the “distinct strengths and vulnerabilities” of physicians when they become patients…due to the “inside knowledge and ability to better navigate the medical system [while also being] uniquely positioned to act as the greatest adversaries to [their] own patient-selves”^73^ (p.2303). She sees “serving others” as the “most powerful strength” whereby “work can be restorative not only to patients but to our own patient-selves”^73^ (p.2304). These observations would seem to apply, at least in part, to other health professionals. In terms of practical implications, one of the major themes of this review is that continuing to work can provide a vital source of meaning. Yet, the work conditions should be adapted to the needs of health professionals who have been afflicted with BC.

A particularly promising avenue could well be rehabilitation per se as part of the RTW trajectory. Several examples from the present review support this contention. Namely, the two physiotherapists with BC, Ameli and Anita, returned to their rehabilitation-related work, which was described as “pleasant…because it was so normal”^22^ (p.320). Concordantly, after completing BC therapy, Dr Julie Silver resuming her work as a rehabilitation specialist, developed a program dedicated to cancer survivorship and wrote a book on that very topic.^62^ Therein, Dr Silver describes the STAR program, an acronym for “Survivorship Training and Rehabilitation”, in which health professionals are appropriately trained to provide cancer survivorship care, as a “best-practices model” (p.11).^62^ The importance of a multi-disciplinary team oriented to the specific vocational needs of women with BC has been underscored. Supportive services can be provided by a range of rehabilitation specialists, including physiotherapists, occupational therapists, occupational health physicians and nurses, and psychological counselors.^17^ Within the rehabilitation framework, the focus could be to explore how health providers as health recipients could best return to healthier work.

An important component of such a framework should certainly be improving work-life balance, to ameliorate ERI, effort-reward imbalance. Indeed, ERI could be most clearly seen in the baseline work conditions of two of the health professionals included in this review.^23,60^ The intrinsic component of ERI, overcommitment, if pronounced, has been associated with increased oxidative stress markers among nurses.^77^ These markers, in turn, are associated with age acceleration,^78^ which, as noted, is linked to heightened BC risk.^6^ Singh and colleagues^77^ emphasize that in order to reduce the associated health risks among nurses, there is a “need for targeted interventions to address this intrinsic stressor, [which is] characterized by difficulty detaching from work and constant rumination” (p.6). As noted for one of the case studies,^60^ shortened workhours, thereby providing more time for recreational physical activity and other healthy social interactions, were key practical ways for the oncology nurse to reduce job overcommitment, while enhancing the meaningfulness/personal rewards of her work. Concordantly, two nurses with BC who participated in a formal exercise program both returned to work. The benefits of the program in promoting RTW were acclaimed by most of the participants in the study.^39^

A participatory action research (PAR) approach has been advocated to design and implement RTW interventions that are both realistic and concordant with the needs of women after BC treatment^79^. It has been emphasized that via PAR, “useful and precise knowledge” can be generated which could inform studies that are both appropriate for the target group and “logistically realistic” (p.3). Indeed, PAR would be particularly appropriate for health professionals with BC.

In examining the studies included in this review in relation to PAR, preliminary suggestions for interventions emerge. Some are based on reported individual experience. For example, from the in-depth follow-up of the 43-year-old nurse, during the course of her treatment and RTW,^23^ a sharp contrast is seen. She describes the pre-BC work conditions: long workhours (70–80 h/week), night call and early morning meetings. With RTW, besides reducing workhours, she explicitly set boundaries, including the need for proper restbreaks for meals, free from unnecessary disturbances. Inadequate, interrupted restbreaks, have been shown to be associated with burnout and poor sleep among health professionals.^9^ Moreover, sufficient, genuine restbreaks are among the most feasible interventions and can be coupled with health-promoting practices.^9^

Reducing workhours and/or diminishing or eliminating nightshift work with RTW were reported to have been requested and/or implemented by several of the health professionals.^26,28,38,52,^^54–57^^,60^ Since prolonged exposure to nightshift work has been associated with BC risk among health professionals,^3,4^ the latter intervention warrants particular attention.

A few of the health professionals requested that other colleagues and staff “step in” with administrative and/or challenging tasks.^25,52,59,60^ Although sometimes eliciting complaints,^52^ overall a spirit of solidarity and support was thereby promoted, an essential component of successful RTW. The special measures needed with lymphedema and other upper arm problems also merit further attention.^30,44,45^

Increased activity within planning, education and policies was quite often reported.^27,46,^^54–56^ This could help minimize direct contact with patients afflicted with end-stage disease, and could be particularly well integrated within the outlined rehabilitation framework.

### Limitations

Nearly all the studies included in this review were observational, mainly narrative. Substantial insights can be gleaned thereby regarding RTW for health professionals with BC. However, robust conclusions cannot be made as to which RTW strategies are most effective and beneficial for this cohort.

A major challenge in conducting this review is the lack of systematic attention to the RTW needs of health professionals with BC. On the other hand, health professionals as care providers have been included in numerous studies on RTW for women with BC. These two roles needed to be “untangled”, via a multi-faceted search strategy and exhaustive examination of a plethora of publications, to finally identify a relatively small number of papers in which at least some information could be garnered on the topic.

The formalities of a systematic review could not be fully implemented. In particular, quality assessment of each study was not a suitable option. Firstly, given that the studies included in this review were primarily qualitative, as pointed out by Tong et al.^35^ “Quality assessment of qualitative research is challenging and contentious” (p.6). More fundamentally, the focus of our review, especially in relation to the RTW studies included in Table 1 differed substantially from the overall aims of those studies. Thus, it would not be appropriate to formally criticize those studies for their lack attention to the specific working conditions and needs of health professionals with BC. Instead, we opted in Table 1 to glean and present the maximal amount of information about those health professionals, and also to indicate where the needed information was lacking. The larger, quantitative studies Refs.^40,44,45^ in Table 1, notwithstanding appropriate statistical analysis and overall methodologic rigor, generally provided even less insight into the specific situation of the health professionals with BC. There were also substantial gaps in the information provided by larger, quantitative publications included in Table 2: Refs.^28,50,51^ addressing RTW among health professionals with various malignancies. On the other hand, rich, multi-faceted insights were provided by many of the qualitative studies in Table 2, as well as in the case studies in Table 3. Taken together, the present review cannot fully meet the requirements of a systematic review, since formal quality appraisal cannot be carried out. The term “systematized review” is therefore more appropriate.

In the present review, none of the health professionals with BC were noted as male. Although primarily a disease of women, a small, yet growing percentage of BC occurs in men.^80^ Obesity as well as occupational exposure to ionizing radiation are among the implicated risk factors for male BC.^80^ A few of the studies in this review included men with malignancies other than BC,^27,39,41,42,46,47^ and from whom some further salient insights can be gleaned. Perhaps most notable was the overriding importance of RTW for preserving their self-image.^41^

The studies that included series of patients with BC were carried out in various countries in Europe, the Middle East, the Far East and Oceania, as well as the U.S. In contrast, however, of the nine cases of female health professionals with BC, all but two explicitly worked in the U.S. Consequently, the generalizability of these case studies is limited.

Notwithstanding these challenges, the present review can be a springboard to inform further efforts to address the special needs, as well as the potentially special contributions of health providers who have been faced with breast cancer when returning to their work.

## Conclusions

Insufficient attention has been given to health professionals who have been afflicted with breast cancer, BC. Special issues arise regarding their returning to work, RTW. Guidelines are vital to help oncologists, oncology nurses, occupational health physicians, occupational therapists, physiotherapists and others, who as health providers address the needs of their colleagues with BC. Job stressors that may contribute to increased BC risk warrant particular scrutiny with RTW for this cohort. A participatory action research, PAR, framework has been advocated to guide RTW strategies for patients with BC.^79^ Such a PAR framework would be particularly suitable to guide intervention studies aimed at identifying the healthiest RTW options for this special cohort.

## Supplemental Material

sj-docx-1-wor-10.1177_10519815251410109 - Supplemental material for Return to work for health professionals with breast cancer as health recipients: A systematized review Supplemental material, sj-docx-1-wor-10.1177_10519815251410109 for Return to work for health professionals with breast cancer as health recipients: A systematized review by Karen Belkić and Brigitte Wilczek in WORK

## List of acronyms

BBE: Bare below the elbow
BC: Breast cancer
BI-RADS: Breast imaging-reporting and data system
ERI: Effort reward imbalance
OSI: Occupational stressor index
PAR: Participatory action research
PTSD: Post-traumatic stress disorder
RTW: Return to work
STAR: Survivorship Training and Rehabilitation
