# Supplementary material for: Return to work for health professionals with breast cancer as health recipients: A systematized review
Source: Work. 2026 Feb 16;84(3):591–619. doi: 10.1177/10519815251410109 (PMC13320139; doi:10.1177/10519815251410109)
Supplement: sj-docx-1-wor-10.1177_10519815251410109 - Supplemental material for Return to work for health professionals with breast cancer as health recipients: A systematized review [file sj-docx-1-wor-10.1177_10519815251410109.docx]

1. Akezaki Y, Nakata E, Kikuuchi M, Tominaga R, Kurokawa H, Hamada M, et al. [Factors associated with return to work of breast cancer patients following axillary lymph node dissection.](https://pubmed-ncbi-nlm-nih-gov.proxy.kib.ki.se/34511470/) Work. 2021;70(1):271-7. doi: 10.3233/WOR-213571. (Table 1)

2. Algeo N, Bennett K, Connolly D. Rehabilitation interventions to support return to work for women with breast cancer: a systematic review and metaanalysis. BMC Cancer. 2021; 21:895. doi.org/10.1186/s12885-021-08613-x

3. Algeo N, Bennett K, Connolly D. Breast cancer survivorship and employment in Ireland: Legislative systems and the return to work of women with breast cancer. Work. 2022; 71(4):927-39. DOI:10.3233/WOR-205044 (Table 1)

4. Algeo N, Bennett K, Connolly D. Prioritising the content and delivery of a work-focused intervention for women with breast cancer using the nominal group technique. Work. 2022;73(4):1337-45. Doi: 10.3233/WOR-211160

5. Allen E-C F, Arroll B. Physician self-disclosure in primary care: a mixed methods study of GPs' attitudes, skills, and behaviour. Br J Gen Pract. 2015; 65(638): e601-8. DOI: 10.3399/bjgp15X686521

6. Amir Z, Wynn P, Whitaker S, Luker K. Cancer survivorship and return to work: UK occupational physician experience. Occup Med. 2009;59(6):390–6. doi:10.1093/occmed/kqn150

7. [Anderson](https://pubmed-ncbi-nlm-nih-gov.proxy.kib.ki.se/?term=Anderson+EA&cauthor_id=35950258) EA, [Anbari](https://pubmed-ncbi-nlm-nih-gov.proxy.kib.ki.se/?term=Anbari+AB&cauthor_id=35950258)  AB, [Sun](https://pubmed-ncbi-nlm-nih-gov.proxy.kib.ki.se/?term=Sun+Y&cauthor_id=35950258) Y,  [Armer](https://pubmed-ncbi-nlm-nih-gov.proxy.kib.ki.se/?term=Armer+JM&cauthor_id=35950258) JM. A multiple case study of Latina breast cancer survivors returning to work with breast cancer-related lymphedema: Adaptation, resilience, and quality of life. Hisp Health Care Int. 2022;20(4):222-30.  doi: 10.1177/15404153221116755

8. Ansmann L, Kowalski C, Ernstmann N, Ommen O, Jung J, Visser A, et al. [Do breast cancer patients receive less support from physicians in German hospitals with high physician workload? A multilevel analysis.](https://pubmed-ncbi-nlm-nih-gov.proxy.kib.ki.se/23890726/) Patient Educ Couns. 2013;93(2):327-34. doi: 10.1016/j.pec.2013.06.016

9. Ansmann L, Wirtz M, Kowalski C, Pfaff H, Visser A, Ernstmann N. [The impact of the hospital work environment on social support from physicians in breast cancer care.](https://pubmed-ncbi-nlm-nih-gov.proxy.kib.ki.se/25082725/) Patient Educ Couns. 2014;96(3):352-60. doi: 10.1016/j.pec.2014.07.016

10. Assogba ELF, Kamga AM, Costaz H, Jankowski C, Dumas A, Roignot P, et al. [What are young women living conditions after breast cancer? Health-related quality of life, sexual and fertility issues, professional reinsertion.](https://pubmed-ncbi-nlm-nih-gov.proxy.kib.ki.se/32545701/) Cancers (Basel). 2020;12(6):1564. doi: 10.3390/cancers12061564

11. Bae KR, So WY, Jang S. Effects of a Post-Traumatic Growth Program on young Korean breast cancer survivors. Healthcare 2023, 11(1), 140. doi.org/10.3390/healthcare11010140

12. Banning M. Employment and breast cancer: a meta-ethnography. Eur J Cancer Care (Engl). 2011; 20(6): 708–19. DOI: 10.1111/j.1365-2354.2011.01291.x

13. Barlow S, Dixey R, Todd J, Taylor V, Carney S, Newell R. ['Abandoned by medicine'? A qualitative study of women's experiences with lymphoedema secondary to cancer, and the implications for care.](https://pubmed-ncbi-nlm-nih-gov.proxy.kib.ki.se/25146257/) Prim Health Care Res Dev. 2014;15(4):452-63. doi: 10.1017/S1463423613000406.

14. Barnes AJ, Robert N, Bradley CJ. [Job attributes, job satisfaction and the return to health after breast cancer diagnosis and treatment.](https://pubmed-ncbi-nlm-nih-gov.proxy.kib.ki.se/24000141/) Psychooncology. 2014;23(2):158-64. doi: 10.1002/pon.3385.

15. Beaver K, Williamson S, Chalmers K. [Telephone follow-up after treatment for breast cancer: views and experiences of patients and specialist breast care nurses.](https://pubmed-ncbi-nlm-nih-gov.proxy.kib.ki.se/20649914/) J Clin Nurs. 2010;19(19-20):2916-24. doi: 10.1111/j.1365-2702.2010.03197.x.

16. Belkić K, Savić C. “Oncology nurse with breast cancer and disturbed sleep.” Job stressors and mental health: A proactive clinical perspective. Toh Tuck (Singapore): World Scientific; 2013, pp. 207-26. (Table 3)

17. Bijker R, Duijts SFA, Smith SN, de Wildt-Liesveld R, Anema JR, Regeer BJ. [Functional impairments and work-related outcomes in breast cancer survivors: A systematic review.](https://pubmed-ncbi-nlm-nih-gov.proxy.kib.ki.se/29086111/) J Occup Rehabil. 2018;28(3):429-51. doi: 10.1007/s10926-017-9736-8.

18. Bilodeau K, Tremblay D, Durand MJ.  [Exploration of return-to-work interventions for breast cancer patients: a scoping review.](https://pubmed-ncbi-nlm-nih-gov.proxy.kib.ki.se/28054145/) Support Care Cancer. 2017;25(6):1993-2007. doi: 10.1007/s00520-016-3526-2.

19. Bilodeau K, Tremblay D, Durand MJ. [Return to work after breast cancer treatments: Rebuilding everything despite feeling "in-between".](https://pubmed-ncbi-nlm-nih-gov.proxy.kib.ki.se/31358250/) Eur J Oncol Nurs. 2019;41:165-72. doi: 10.1016/j.ejon.2019.06.004. Although explicitly included in this study of women with breast cancer vis-à-vis return to work, no specific information can be gleaned about the woman who had been employed within the health care sector.

20. Bilodeau K, Tremblay D, Durand MJ.  [Gaps and delays in survivorship care in the return-to-work pathway for survivors of breast cancer-a qualitative study.](https://pubmed-ncbi-nlm-nih-gov.proxy.kib.ki.se/31285687/) Curr Oncol. 2019;26(3):e414-7. doi: 10.3747/co.26.4787.

21. Bilodeau K, Gouin MM, Lecours A, Lederer V, Durand MJ, Kilpatrick K, et al. [Acceptability and feasibility of a return-to-work intervention for posttreatment breast cancer survivors: Protocol for a co-design and development study.](https://pubmed-ncbi-nlm-nih-gov.proxy.kib.ki.se/35451972/) JMIR Res Protoc. 2022;11(4):e37009. doi: 10.2196/37009.

22. Björneklett HG, Rosenblad A, Lindemalm C, Ojutkangas ML, Letocha H, Strang P, et al. A randomized controlled trial of support group intervention after breast cancer treatment: results on sick leave, health care utilization and health economy. Acta Oncol. 2013;52(1):38-47.doi.org/10.1016/j.jpsychores.2012.11.005

23. Blinder V, Murphy M, Vahdat L, Gold H, de Melo-Martin I, Hayes MK, et al. Employment after a breast cancer diagnosis: A qualitative study of ethnically diverse urban women. J Community Health 2012; 37(4): 763-72. DOI 10.1007/s10900-011-9509-9

24. Bogale N, Balta B, Demissie G, Geleta D, Rakoff M, Anderson B, et al. [Exploring challenges related to breast cancer to identify opportunities for advocacy in Hawassa City, Southern Ethiopia: A community-based, qualitative study.](https://pubmed-ncbi-nlm-nih-gov.proxy.kib.ki.se/37972329/) JCO Glob Oncol. 2023;9:e2300137. doi: 10.1200/GO.23.00137

25. Böttcher H, Steimann M, Rotsch M, Zurborn K-H, Koch U, Bergelt C. Occupational stress and its association with early retirement and subjective need for occupational rehabilitation in cancer patients. Psychooncology. 2013; 22(8): 1807–14. DOI: 10.1002/pon.3224

26. Böttcher H, Steimann M, Ullrich A, Rotsch M, Zurborn K-H, Koch U, et al. Work-related predictors of not returning to work after inpatient rehabilitation in cancer patients. Acta Oncol. 2013; 52(6): 1067–75. DOI: 10.3109/0284186X.2013.792991

27. Bouknight RR, Bradley CJ, Luo Z Correlates of return to work for breast cancer survivors. J Clin Oncol. 2006; 24(3): 345-52. DOI: 10.1200/JCO.2004.00.4929

28. Bouzgarrou L, Laajili H, Slama MA, Omrane A, Ben Azia L, Kammoun S. Breast cancer survivors' returning to work: An observational study among active women, two years after diagnosis. Curr Womens Health Rev. 2024; 20 (1):doi 10.2174/1573404819666230119154625

29. Brearley S, Stamataki Z, Addington-Hall J, Foster C, Hodges L, Jarrett N, et al. The physical and practical problems experienced by cancer survivors: A rapid review and synthesis of the literature. Eur J Oncol Nurs.  2011; 15(3): 204−12. doi:10.1016/j.ejon.2011.02.005

30. Broc G, Carretie J, Rouat S, Guittard L, Péron J, Fervers B, et al. Design and production of a patient guide to support return to work after breast cancer: An application of intervention mapping. Psycho-Oncologie. 2023; 17(3): 167-179. FRENCH

31. Brophy JT, Keith MM, Gorey KM, Luginaah I, Laukkanen E, Hellyer D, et al. [Occupation and breast cancer: A Canadian case-control study.](https://pubmed-ncbi-nlm-nih-gov.proxy.kib.ki.se/17119253/) Ann N Y Acad Sci. 2006;1076:765-77. doi: 10.1196/annals.1371.019

32. Bulut A, Bulut A. [Knowledge, attitudes and behaviors of primary health care nurses and midwives in breast cancer early diagnosis applications.](https://pubmed-ncbi-nlm-nih-gov.proxy.kib.ki.se/28331367/) Breast Cancer (Dove Med Press). 2017;9:163-9. doi: 10.2147/BCTT.S126124

33. Bush NJ. Post-traumatic stress disorder related to the cancer experience. Oncol Nurs Forum. 2009; 36(4): 395–400. [doi.org/10.1188/09.ONF.395-400](https://doi.org/10.1188/09.ONF.395-400) (Table 3)

34. Butow P, Laidsaar-Powell R, Konings S, Yi Shing Lim C, Koczwara B. Return to work after a cancer diagnosis: a meta-review of reviews and a meta-synthesis of recent qualitative studies. J Cancer Surviv. 2020;14(2):114- 34. [doi.org/10.1007/s11764-019-00828-z](https://doi.org/10.1007/s11764-019-00828-z)

35. Calvio L, Peugeot M, Bruns GL, Todd BL, Feuerstein M. Measures of cognitive function and work in occupationally active breast cancer survivors. J Occup Environ Med. 2010;52:219–27. DOI: 10.1097/JOM.0b013e3181d0bef7

36. Campagna M, Loscerbo R, Pilia I, Meloni F. [Return to work of breast cancer survivors: Perspectives and challenges for occupational physicians.](https://pubmed-ncbi-nlm-nih-gov.proxy.kib.ki.se/32033165/) Cancers (Basel). 2020;12(2):355. doi:10.3390/cancers12020355

37. Carlsen K, Jung Jensen A, Rugulies R, Christensen J , Envold Bidstrup P, Johansen C, et al. Self-reported work ability in long-term breast cancer survivors. A population-based questionnaire study in Denmark. Acta Oncol. 2013; 52(2): 423– 9. DOI: 10.3109/0284186X.2012.744877

38. Carlson LE, Subnis UB, Piedalue KL, Vallerand J, Speca M, Lupichuk S, et al. [The ONE-MIND Study: Rationale and protocol for assessing the effects of ONlinE MINDfulness-based cancer recovery for the prevention of fatigue and other common side effects during chemotherapy.](https://pubmed-ncbi-nlm-nih-gov.proxy.kib.ki.se/31056784/) Eur J Cancer Care (Engl). 2019;28(4):e13074. DOI: 10.1111/ecc.13074

39. Caron M, Durand M-J, Tremblay D. Perceptions of breast cancer survivors on the supporting practices of their supervisors in the return-to-work process: A qualitative descriptive study. J Occup Rehabil. 2018;28:89-96. Doi: 10.1007/s10926-017-9698-x

40. Castro-Frenzel K. [Physician-as-patient-vulnerabilities and strengths.](https://pubmed-ncbi-nlm-nih-gov.proxy.kib.ki.se/36538307/) JAMA. 2022; 328(23):2303-4. doi: 10.1001/jama.2022.21859

41. Cheng ASK, Liu X, Ng PHF, Kwok CTT, Zeng Y, Feuerstein M. [Breast cancer application protocol: A randomised controlled trial to evaluate a self-management app for breast cancer survivors.](https://pubmed-ncbi-nlm-nih-gov.proxy.kib.ki.se/32624468/) BMJ Open. 2020;10(7):e034655. doi: 10.1136/bmjopen-2019-034655

42. Cheng ASK, Lee S, Li N, Tsang S, Zeng Y. [Chinese translation and cross-cultural adaptation of the return-to-work self-efficacy scale among Chinese female breast cancer survivors.](https://pubmed-ncbi-nlm-nih-gov.proxy.kib.ki.se/36901236/) Int J Environ Res Public Health. 2023;20(5):4225. doi: 10.3390/ijerph20054225

43. Cohen M, Yagil D, Carel R. A multidisciplinary working model for promoting return to work of cancer survivors. Support Care Cancer 2021;29(9):5151-60. doi.org/10.1007/s00520-021-06074-3

44. Consalvo KE, Piscitelli LD, Williamson L Policarpo G, Englander M, Lyons K, et al. Treating one of our own. Clin J Oncol Nurs. 2007; 11(2):227–31. DOI: 10.1188/07.CJON.227-231 (Table 3)

45. Cooper AF, Hankins M, Rixon L, Grunfeld EA. Distinct work-related, clinical and psychological factors predict return to work following treatment in four different cancer types. Psychooncology. 2013; 22(3):659-67. DOI: 10.1002/pon.3049

46. Corbière M, Rabouin D, Negrini A, Mazaniello-Chézol M, Sideris L, Prady C, et al. [Validation of the Return-to-Work Obstacles and Self-Efficacy Scale for women on sick leave due to breast cancer (ROSES-BC).](https://pubmed-ncbi-nlm-nih-gov.proxy.kib.ki.se/38311709/) J Occup Rehabil. 2024;34(4):818-31. doi: 10.1007/s10926-023-10169-5

47. De Blasi G, Bouteyre E, Rollin L. Giving up work after cancer: An exploratory qualitative study of three clinical cases. Work. 2018; 60 (1); 105-5. DOI:10.3233/WOR-182712

48. de Boer AGEM, Frings-Dresen MHW. Employment and the common cancers: Return to work of cancer survivors. Occup Med (London). 2009;59:378–80. doi:10.1093/occmed/kqp087

49. de Boer AG, Taskila T, Tamminga SJ, Frings-Dresen MH, Feuerstein M, Verbeek JH. Interventions to enhance return-to-work for cancer patients. Cochrane Database Syst Rev. 2011;(2):CD007569. DOI: 10.1002/14651858.CD007569.pub2

50. de Boer AGEM, Taskila TK, Tamminga SJ, Feuerstein M, Frings-Dresen MHW, Verbeek JH. Interventions to enhance return-to-work for cancer patients. Cochrane Database Syst Rev. 2015; (9): CD007569. DOI: 10.1002/14651858.CD007569.pub3

51. de Boer AG, De Wind A, Coenen P, Van Ommen F, Greidanus M, Zegers AD, et al. Cancer survivors and adverse work outcomes: associated factors and supportive interventions. Br Med Bull. 2023;145:60-70. [doi.org/10.1093/bmb/ldac028](https://doi.org/10.1093/bmb/ldac028)

52. de Groef A, Van der Gucht E, Devoogdt N, Smeets A, Bernar K, Morlion B, et al. [Returning to work after breast cancer surgery: A randomised controlled trial on the effect of pain neuroscience education.](https://pubmed-ncbi-nlm-nih-gov.proxy.kib.ki.se/37171771/) J Occup Rehabil. 2023;33(4):757-65. doi: 10.1007/s10926-023-10103-9

53. Deimling, GT, Pappada H, Ye M, Nalepa E, Ciaralli S, Phelps E, et al. Factors affecting perceptions of disability and self-rated health among older adult, long-term cancer survivors. J Aging Health 2019; 31(4): 667-84. DOI: 10.1177/0898264317745745

54. Del Piccolo L, Mazzi MA, Mascanzoni A, Lonardi M, De Felice M, Danzi OP, et al. [Factors related to the expression of emotions by early-stage breast cancer patients.](https://pubmed-ncbi-nlm-nih-gov.proxy.kib.ki.se/30967297/) Patient Educ Couns. 2019;102(10):1767-73. doi: 10.1016/j.pec.2019.04.002

55. De Marco RF, Picard C, Agretelis J. Nurse experiences as cancer survivors: part I-personal. Oncol Nurs Forum. 2004;31(3):523-30. doi: 10.1188/04.ONF.523-530. (Table 2)

56. Désiron HA, Donceel P, de Rijk A, Van Hoof E. A conceptual-practice model for occupational therapy to facilitate return to work in breast cancer patients. J Occup Rehabil. 2013;23(4):516-26. DOI 10.1007/s10926-013-9427-z

57. Désiron HA, Crutzen R, Godderis L, Van Hoof E, de Rijk A. [Bridging health care and the workplace: Formulation of a return-to-work intervention for breast cancer patients using an intervention mapping approach.](https://pubmed-ncbi-nlm-nih-gov.proxy.kib.ki.se/26728492/) J Occup Rehabil. 2016;26(3):350-65. doi: 10.1007/s10926-015-9620-3

58. Désiron H, Simons B, Spooren A, Camut S, Van de Velde D, Otte T, et al.  [Practice-based evidence to support return to work in cancer patients.](https://pubmed-ncbi-nlm-nih-gov.proxy.kib.ki.se/36189048/) Front Rehabil Sci. 2022;3:819369. doi: 10.3389/fresc.2022.819369

59. Dias M, Zomkowski K, Silva Michels FA, Sperandio FF. Breast cancer surgery effect on professional activities. Cad Bras Ter Ocup, São Carlos. 2017; 25(2): 325-32. PORTUGUESE

60. Drageset S, Lindstrøm TC, Underlid K. ["I just have to move on": Women's coping experiences and reflections following their first year after primary breast cancer surgery.](https://pubmed-ncbi-nlm-nih-gov.proxy.kib.ki.se/26521054/) Eur J Oncol Nurs. 2016;21:205-11. doi: 10.1016/j.ejon.2015.10.005

61. Drolet M, Maunsell E, Brisson J, Brisson C, Masse B, Deschenes L. Not working 3 years after breast cancer: Predictors in a population-based study. J Clin Oncol. 2005;23(33):8305–12. DOI: 10.1200/JCO.2005.09.500

62. Dugan AG, Decker RE, Namazi S, Cavallari JM, Bellizzi KM, Blank TO, et al. [Perceptions of clinical support for employed breast cancer survivors managing work and health challenges.](https://pubmed-ncbi-nlm-nih-gov.proxy.kib.ki.se/33405056/) J Cancer Surviv. 2021;15(6):890-905. doi: 10.1007/s11764-020-00982-9

63. Edward K-L, Giandinoto J-A, McFarland J. Analysis of the experiences of nurses who return to nursing after cancer. Br J Nurs. 2017; 26(21):1170–5. (Table 2)

64. Fagundo-Rivera J, Gómez-Salgado J, García-Iglesias JJ, Allande-Cussó R, Ortega-Moreno M, Ruiz-Frutos C. [Work, family and nurses perception about their own health: Relationship with breast cancer and shift work.](https://pubmed-ncbi-nlm-nih-gov.proxy.kib.ki.se/34075016/) Rev Esp Salud Publica. 2021;95:e202106078. SPANISH

65. Fassier JB, Guittard L, Fervers B, Rouat S, Sarnin P, Carretier J, et al.  [Using intervention mapping to facilitate and sustain return-to work after breast cancer: protocol for the FASTRACS multicentre randomized controlled trial.](https://pubmed-ncbi-nlm-nih-gov.proxy.kib.ki.se/39237867/) BMC Cancer. 2024;24(1):1107. doi: 10.1186/s12885-024-12796-4

66. Fauser D, Wienert J, Zomorodbakhsch B, Schmielau J, Biester I, Krüger HU, et al. Work-related medical rehabilitation in cancer: A cluster-randomized multicenter study. Dtsch Arztebl Int. 2019;116(35-36):592-9. DOI: 10.3238/arztebl.2019.0592

67. Feuerstein M, Todd BL, Moskowitz MC, Bruns GL, Stoler Mallori R, Nassif T, et al. Work in cancer survivors: A model for practice and research. J Cancer Surviv. 2010;4(4): 415-37. DOI 10.1007/s11764-010-0154-6

68. Finkelstein EA, Malkin JD, Baid D, Alqunaibet A, Mahdi K, Al-Thani MBH, et al. [The impact of seven major noncommunicable diseases on direct medical costs, absenteeism, and presenteeism in Gulf Cooperation Council countries.](https://pubmed-ncbi-nlm-nih-gov.proxy.kib.ki.se/34138664/) J Med Econ. 2021;24(1):828-34. doi: 10.1080/13696998.2021

69. Franco G. [Occupation and breast cancer: Fitness for work is an aspect that needs to be addressed.](https://pubmed-ncbi-nlm-nih-gov.proxy.kib.ki.se/23789515/) Med Lav. 2013;104(2):87-92.

70. Fromme EK, Hebert RS, Carrese JA. Self-doctoring: A qualitative study of physicians with cancer. J Fam Pract 2004; 53(4): 299-306. (Table 2)

71. Ganem G, Antoine EC, Touboul C, Naman H, Dohollou N, Facchini T, et al. [Maintaining professional activity during breast cancer treatment.](https://pubmed-ncbi-nlm-nih-gov.proxy.kib.ki.se/26891443/) Eur J Cancer Care (Engl). 2016;25(3):458-65. DOI: 10.1111/ecc.12460

72. Ganz PA, Yip CH, Gralow JR, Distelhorst SR, Albain KS, Andersen BL, et al. Supportive care after curative treatment for breast cancer (survivorship care): Resource allocations in lowand middle-income countries. A Breast Health Global Initiative 2013 consensus statement. Breast. 2013;22(5): 606-15. doi.org/10.1016/j.breast.2013.07.049

73. Ghasempour M, Shabanloei R, Rahmani A, Jafarabadi MA, Abri F, Khajehgoodari M. The relation of readiness for return to work and return to work among Iranian cancer survivors. J Cancer Educ. 2020; 35 (6): 1237-42. doi.org/10.1007/s13187-019-01588-1

74. Giese-Davis J, Bliss-Isberg C, Wittenberg L, White J, Star P, Zhong L, et al. Peercounseling for women newly diagnosed with breast cancer: A randomized community/ research collaboration trial. Cancer. 2016;122(15):2408-17. doi:10.1002/cncr.30036

75. Girgis A, Stacey F, Lee T, Black D, Kilbreath S. [Priorities for women with lymphoedema after treatment for breast cancer: population based cohort study.](https://pubmed-ncbi-nlm-nih-gov.proxy.kib.ki.se/21693532/) BMJ. 2011;342:d3442. doi: 10.1136/bmj.d3442

76. [Gonzalez BD, Grandner MA, Caminiti CB, Hui SA. Cancer survivors in the workplace: Sleep disturbance mediates the impact of cancer on healthcare expenditures and work absenteeism.](https://pubmed-ncbi-nlm-nih-gov.proxy.kib.ki.se/29869719/) Support Care Cancer. 2018;26(12):4049-55. doi.org/10.1007/s00520-018-4272-4

77. Goss C, Leverment IM, de Bono AM. [Breast cancer and work outcomes in health care workers.](https://pubmed-ncbi-nlm-nih-gov.proxy.kib.ki.se/25149119/) Occup Med (Lond). 2014;64(8):635-7. doi:10.1093/occmed/kqu122 (Table 2)

78. Groeneveld I F, de Boer Angela GEM,Frings-Dresen MHW. Physical exercise and return to work: Cancer survivors’ experiences. J Cancer Surviv. 2013; 7(2):237-46. DOI 10.1007/s11764-012-0264-4 (Table 1)

79. Grunfeld EA, Schumacher L, Armaou M, Woods PL, Rolf P, Sutton AJ, et al. Feasibility randomised controlled trial of a guided workbook intervention to support work-related goals among cancer survivors in the UK. BMJ Open. 2019;9(1):e022746. doi:10.1136/bmjopen-2018-022746

80. Guittard M, Capitain O, Guittard E, Roquelaure Y, Petit A. Factors influencing return to work and job retention after breast cancer. Archives des Maladies Professionnelles et de L’Environnement. 2016; 77 (2): 157-64. FRENCH

81. Guseva Canu I, Bovio N, Arveux P, Bulliard JL, Fournier E, Germann S, et al. [Breast cancer and occupation: Non-parametric and parametric net survival analyses among Swiss women (1990-2014).](https://pubmed-ncbi-nlm-nih-gov.proxy.kib.ki.se/37089493/) Front Public Health. 2023;11:1129708. doi: 10.3389/fpubh.2023.1129708

82. Hansen JA, Feuerstein M, Calvio LC, Olsen CH. Breast cancer survivors at work. J Occup Environ Med. 2008;50(7):777–84. DOI: 10.1097/JOM.0b013e318165159e

83. Hedayati E, Johnsson A, Alinaghizadeh H, Schedin A, Nyman H, Albertsson M. Cognitive, psychosocial, somatic and treatment factors predicting return to work after breast cancer treatment. Scand J Caring Sci. 2013; 27; 380–7. doi: 10.1111/j.1471-6712.2012.01046.x

84. Heidkamp P, Hiltrop K, Breidenbach C, Kowalski C, Pfaff H, Geiser F, et al. [Coping with breast cancer during medical and occupational rehabilitation: A qualitative study of strategies and contextual factors.](https://pubmed-ncbi-nlm-nih-gov.proxy.kib.ki.se/38504261/) BMC Womens Health. 2024;24(1):183. doi: 10.1186/s12905-024-03012-3

85. Hequet D, Hamy AS, Girard N, Laas E, Coussy F, Rouzier R, et al. [Variation over time of the factors influencing return to work and work capacities after a diagnosis of breast cancer: a study on the behalf of the Seintinelles research network.](https://pubmed-ncbi-nlm-nih-gov.proxy.kib.ki.se/35396963/) Support Care Cancer. 2022;30(7):5991-9. doi: 10.1007/s00520-022-07000-x

86. Heuser C, Halbach S, Kowalski C, Enders A, Pfaff H, Ernstmann N.  [Sociodemographic and disease-related determinants of return to work among women with breast cancer: a German longitudinal cohort study.](https://pubmed-ncbi-nlm-nih-gov.proxy.kib.ki.se/30594181/) BMC Health Serv Res. 2018;18(1):1000. doi: 10.1186/s12913-018-3768-4

87. Hiltrop K, Heidkamp P, Kowalski C, Ernstmann N. [Breast cancer patients' return to work (B-CARE): protocol of a longitudinal mixed-methods study aiming to explore medical and occupational rehabilitation of patients with breast cancer in Germany.](https://pubmed-ncbi-nlm-nih-gov.proxy.kib.ki.se/31874892/) BMJ Open. 2019;9(12):e033533. doi: 10.1136/bmjopen-2019-033533

88. Hiltrop K, Heidkamp P, Breidenbach C, Kowalski C, Enders A, Pfaff H, et al.. [Involuntariness of job changes is related to less satisfaction with occupational development in long-term breast cancer survivors.](https://pubmed-ncbi-nlm-nih-gov.proxy.kib.ki.se/33907994/) J Cancer Surviv. 2022 ;16(2):397-407. doi: 10.1007/s11764-021-01035-5

89. Hoefsmit N, Houkes I, Nijhuis F. Environmental and personal factors that support early return-to-work: A qualitative study using the ICF as a framework. Work. 2014; 48(2): 203–15. DOI 10.3233/WOR-131657

90. [Holmberg](https://pubmed-ncbi-nlm-nih-gov.proxy.kib.ki.se/?term=Holmberg+C&cauthor_id=23448954) C. No one sees the fear: Becoming diseased before becoming ill--being diagnosed with breast cancer. Cancer Nurs. 2014;37(3):175-83.  doi: 10.1097/NCC.0b013e318281395e

91. Hou W, Li Q, Liu X, Zeng Y, Cheng AS. [Exploring the employment readiness and return to work status of breast cancer patients and related factors.](https://pubmed-ncbi-nlm-nih-gov.proxy.kib.ki.se/34631993/) Int J Nurs Sci. 2021;8(4):426-31. doi.org/10.1016/j.ijnss.2021.09.001

92. Hubbard G, Gray NM, Ayansina D, Evans JM, Kyle RG. Case management vocational rehabilitation for women with breast cancer after surgery: A feasibility study incorporating a pilot randomised controlled trial. Trials. 2013; 14:175. doi:10.1186/1745-6215-14-175 Although explicitly included in this study of women with breast cancer vis-à-vis return to work, no specific information can be gleaned about the women employed within the health care sector.

93. Islam T, Dahlui M, Majid HA, Nahar AM, Mohd Taib NA, Su TT. Factors associated with return to work of breast cancer survivors: A systematic review. BMC Public Health. 2014;14(3):S8. doi:10.1186/1471-2458-14-S3-S8

94. Jin JH, Lee EJ. Effect of workplace spirituality on quality of work life of nurse cancer survivors in South Korea. Asia Pacific J Oncol Nurs 2020a; *7*(4): 346–54.

doi.org/10.4103/apjon.apjon_36_20 (Table 2)

95. Jin JH, Lee EJ. Factors affecting quality of work life in a sample of cancer survivor female nurses. Medicina (Kaunas) 2020; 56(12):721. doi:10.3390/medicina56120721 (Table 2)

96. Johnsson A, Fornander T, Olsson M, Nystedt M, Johansson H, Rutqvist LE. Factors associated with return to work after breast cancer treatment. Acta Oncol. 2007; 46(1): 90-6. DOI: 10.1080/02841860600857318

97. Johnsson A, Fornander T, Rutqvist L-E, Vaez M, Alexanderson K, Olsson M. Predictors of return to work ten months after primary breast cancer surgery. Acta Oncol. 2009; 48(1): 93-8. DOI: 10.1080/02841860802477899

98. Johnsson A, Fornander T, Rutqvist LE, Olsson M. Factors influencing return to work: a narrative study of women treated for breast cancer. Eur J Cancer Care (Engl).  2010; 19(3): 317–23. DOI: 10.1111/j.1365-2354.2008.01043.x (Table 1)

99. Johnsson A, Fornander T, Rutqvist LE, Olsson M. Work status and life changes in the first year after breast cancer diagnosis. Work. 2011;38(4):337-46. DOI 10.3233/WOR20111137

100. Jones JM, Howell D, Longo C, Olson K, Bedard P, Amir E, et al. [The association of cancer-related fatigue on the social, vocational and healthcare-related dimensions of cancer survivorship.](https://pubmed-ncbi-nlm-nih-gov.proxy.kib.ki.se/37644355/) J Cancer Surviv. 2023 Aug 30. doi: 10.1007/s11764-023-01451-9 (online ahead of print)

101. Kaelin C. When a breast cancer expert gets cancer. Harv Womens Health Watch. 2005; 12: 4-6. (Table 3)

102. Kaelin C. Living through breast cancer. New York: McGraw-Hill; 2005. (Table 3)

103. Kamal KM, Covvey JR, Dashputre A, Ghosh S, Shah S, Bhosle M, et al. [A systematic review of the effect of cancer treatment on work productivity of patients and caregivers.](https://pubmed-ncbi-nlm-nih-gov.proxy.kib.ki.se/28125370/) J Manag Care Spec Pharm. 2017;23(2):136-62. doi: 10.18553/jmcp.2017.23.2.136

104. Kang D, Bae KR, Kim HY, Ahn Y, Kim N, Shim Y, et al. [Changes in working status after cancer diagnosis and socio-demographic, clinical, work-related, and psychological factors associated with it.](https://pubmed-ncbi-nlm-nih-gov.proxy.kib.ki.se/36008854/) BMC Cancer. 2022;22(1):917. doi: 10.1186/s12885-022-10013-8

105. Kay M, Mitchell G, Clavarino A, Doust J. [Doctors as patients: A systematic review of doctors' health access and the barriers they experience.](https://pubmed-ncbi-nlm-nih-gov.proxy.kib.ki.se/18611318/) Br J Gen Pract. 2008;58(552):501-8. doi: 10.3399/bjgp08X319486

106. Kay M, Mitchell G, Clavarino A. [What doctors want? A consultation method when the patient is a doctor.](https://pubmed-ncbi-nlm-nih-gov.proxy.kib.ki.se/21133299/) Aust J Prim Health. 2010;16(1):52-9. doi: 10.1071/py09052

107. Kennedy F, Haslam C, Munir F, Pryce J. [Returning to work following cancer: A qualitative exploratory study into the experience of returning to work following cancer.](https://pubmed-ncbi-nlm-nih-gov.proxy.kib.ki.se/17227349/) Eur J Cancer Care (Engl). 2007;16(1):17-25. doi: 10.1111/j.1365-2354.2007.00729.x (Table 1)

108. Kennedy F, Harcourt D, Rumsey N. [The challenge of being diagnosed and treated for ductal carcinoma in situ (DCIS).](https://pubmed-ncbi-nlm-nih-gov.proxy.kib.ki.se/18023255/) Eur J Oncol Nurs. 2008;12(2):103-11. doi: 10.1016/j.ejon.2007.09.007

109. Kennedy F, Harcourt D, Rumsey N.  [Perceptions of ductal carcinoma in situ (DCIS) among UK health professionals.](https://pubmed-ncbi-nlm-nih-gov.proxy.kib.ki.se/19231188/) Breast. 2009;18(2):89-93. doi: 10.1016/j.breast.2009.01.004

110. Kennedy F, Harcourt D, Rumsey N, White P.  [The psychosocial impact of ductal carcinoma in situ (DCIS): a longitudinal prospective study.](https://pubmed-ncbi-nlm-nih-gov.proxy.kib.ki.se/20413310/) Breast. 2010;19(5):382-7. doi: 10.1016/j.breast.2010.03.024

111. Kobayashi M, Sezai I, Ishikawa T, Masujima M. Psychological and educational support for cancer patients who return to work: A scoping review. Work 2022; 73(1): 291–300. DOI:10.3233/WOR-205326

112. Kong YC, Wong LP, Ng CW, Taib NA, Bhoo-Pathy NT, Yusof MM, et al. [Understanding the financial needs following diagnosis of breast cancer in a setting with universal health coverage.](https://pubmed-ncbi-nlm-nih-gov.proxy.kib.ki.se/31922332/) Oncologist. 2020;25(6):497-504. doi: 10.1634/theoncologist.2019-0426

113. Lagad A, Hodgkinson K, Newton-John TRO. Is ignorance bliss, or is knowledge power? When cancer healthcare professionals become cancer patients. Eur J Cancer Care (Engl). 2019; 28(4): e13066. https://doi.org/10. 1111/ecc.13066 (Table 2)

114. Lamort-Bouché M, Péron J, Broc G, Kochan A, Jordan C, Letrilliart L, et al. [Breast cancer specialists' perspective on their role in their patients' return to work: A qualitative study.](https://pubmed-ncbi-nlm-nih-gov.proxy.kib.ki.se/31433060/) Scand J Work Environ 2020;46(2):177-87. doi:10.5271/sjweh.3847

115. Lange M, Lequesne J, Dumas A, Clin B, Vaz-Luis I, Pistilli B, et al  [Cognition and return to work status 2 years after breast cancer diagnosis.](https://pubmed-ncbi-nlm-nih-gov.proxy.kib.ki.se/39158915/) JAMA Netw Open. 2024;7(8):e2427576. doi: 10.1001/jamanetworkopen.2024.27576

116. Lewis J, Chapparo C, Mackenzie L, Ranka J. Work after breast cancer: Identification of cognitive difficulties using the Perceive, Recall, Plan, and Perform (PRPP) System of Task Analysis. Br J Occup Ther. 2016; 79(5): 323-32. DOI: 10.1177/0308022616639983

[117. Lewis J, Mackenzie L. Cognitive changes after breast cancer: a scoping review to identify problems encountered by women when returning to work. Disabil Rehabil. 2022;44(18):5310-5328.  doi: 10.1080/09638288.2021.1919216](https://research-ebsco-com.proxy.kib.ki.se/c/77ure3/search/details/4yfc6w3k7r?db=cin20%2Cldpsy%2Cpdh%2Cpsyh%2Ccmedm%2Clxh%2C8gh%2Ctrh%2Cnlebk&isDashboardExpanded=true&limiters=None&q=%28%28physicians%20or%20doctors%20or%20clinicians%20or%20nurses%20or%20physical%20therapists%20or%20occupational%20therapists%20or%20respiratory%20therapists%20or%20midwives%20or%20health%20professionals%29%29%20AND%20%28breast%20cancer%29%20AND%20%28return%20to%20work%29)

118. Lilliehorn S, Hamberg K, A. Kero, P. Salander. Meaning of work and the returning process after breast cancer: a longitudinal study of 56 women. Scand J Caring Sci. 2013; 27; 267–74. doi: 10.1111/j.1471-6712.2012.01026.x

119. Lindbohm M-L, Kuosma E, Taskila T, Hietanen P, Carlsen K, Gudbergsson S, et al. Cancer as the cause of changes in work situation (a NOCWO study). Psychooncology 2011; 20: 805–12. DOI: 10.1002/pon.1797

120. Liu S, Wang F, Yang Q, Wang Q, Feng D, Chen Y, et al. [Work productivity loss in breast cancer survivors and its effects on quality of life.](https://pubmed-ncbi-nlm-nih-gov.proxy.kib.ki.se/34511524/) Work. 2021;70(1):199-207. doi: 10.3233/WOR-213565

121. Liu XY, Chen C, Wu Q, Ji Q, Zhu P. Dilemma: Disclosure experience among young female breast cancer survivors in China. J Adolesc Young Adult Oncol. 2022; 11 (5); 486-92. DOI: 10.1089/jayao.2021.0124

122. MacLennan S, Cox T, Murdoch S, Eatough V. An interpretative phenomenological analysis of the meaning of work to women living with breast cancer. Chronic Illn. 2022;18(3):503-16. Doi: 10.1177/1742395320987883 Although explicitly included in this study of women with breast cancer vis-à-vis return to work, no specific information can be gleaned about the women employed within the health care sector.

123. Magnavita N, Di Prinzio RR, Meraglia I, Vacca ME, Arnesano G, Merella M, et al. [Supporting return to work after breast cancer: A mixed method study.](https://pubmed-ncbi-nlm-nih-gov.proxy.kib.ki.se/37628540/) Healthcare (Basel). 2023;11(16):2343. doi.org/10.3390/healthcare11162343

124. Mao B, Shen Y, Chen Y, Zhou P, Pan Y. Experiences of healthcare professionals returning to work post breast cancer diagnosis in China: A descriptive qualitative study. Sci Rep. 2025; 15(1):1938 doi.org/10.1038/s41598-024-82893-8 (Table 2)

125. Mapi NS A nurse’s journey with cancer. Asia Pacific J Oncol Nurs. 2018; *5*(3): 290–5. https://doi.org/10.4103/ apjon.apjon_10_18 (Table 3)

126. Marinas-Sanz R, Iguacel I, Maqueda J, Mínguez L, Alquézar P, Andrés R, et al. [Facilitating factors and barriers in the return to work of working women survivors of breast cancer: A qualitative study.](https://pubmed-ncbi-nlm-nih-gov.proxy.kib.ki.se/36765831/) Cancers (Basel). 2023;15(3):874. doi: 10.3390/cancers15030874

127. Maunsell E, Brisson C, Dubois L, Lauzier S, Fraser A [Work problems after breast cancer: an exploratory qualitative study.](https://pubmed-ncbi-nlm-nih-gov.proxy.kib.ki.se/10607979/) Psychooncology. 1999;8(6):467-73. doi: 10.1002

128. Maunsell E, Drolet M, Brisson J, Brisson C, Mâsse B, Deschênes L. Work situation after breast cancer: Results from a population-based study. J Natl Cancer Inst. 2004; 96(24): 1813-22. DOI: 10.1093/jnci/djh335

129. McCorkle R. Cancer nurse as cancer survivor. Cancer Nurs. (2012; 35(3), 245–6. [https://doi.org/10.1097/NCC.0b013e3 1824d2b71](https://doi.org/10.1097/NCC.0b013e3%201824d2b71) (Table 3)

130. McGregor S. Surviving cancer without compromising aspirations. Eur J Oncol Nurs. 2011; 15(3): 201−3. doi:10.1016/j.ejon.2011.03.009

131. Mehnert A; de Boer A, Feuerstein M. Employment challenges for cancer survivors. Cancer 2013; 119(11 Suppl):2151-9. DOI: 10.1002/cncr.28067

132. Mehnert A, Koch U. Work satisfaction and quality of life in cancer survivors in the first year after oncological rehabilitation. Work 2013; 46(4): 407-15. DOI 10.3233/WOR-131676

133. Melisko ME, Gradishar WJ, Moy B.  [Issues in breast cancer survivorship: optimal care, bone health, and lifestyle modifications.](https://pubmed-ncbi-nlm-nih-gov.proxy.kib.ki.se/27249727/) Am Soc Clin Oncol Educ Book. 2016;35:e22-9. doi: 10.1200/EDBK_159203

134. Melnyk H, Djukic M, Merriman J, Vaughan Dickson V. [An integrative review: Women's psychosocial vulnerability in relation to paid work after a breast cancer diagnosis.](https://pubmed-ncbi-nlm-nih-gov.proxy.kib.ki.se/33368563/) J Adv Nurs. 2021;77(5):2144-54. DOI: 10.1111/jan.14730

135. Mitchell T. Both sides of the couch: A qualitative exploration of the experiences of female healthcare professionals returning to work after treatment for cancer. Eur J Cancer Care (Engl). 2015; 24(6): 840–53. https://doi.org/10.1111/ ECC.12308 (Table 2)

136. Mock V.  [Breast cancer and fatigue: issues for the workplace.](https://pubmed-ncbi-nlm-nih-gov.proxy.kib.ki.se/9923203/) AAOHN J. 1998;46(9):425-31.

137. Moskowitz MC, Feuerstein M, Todd BL. Job stress and physical activity related to elevated symptom clusters in breast cancer survivors at work. J Occup Environ Med.  2013; 55(1): 93−8. DOI: 10.1097/JOM.0b013e31826eef97

138. Munir F, Yarker J, McDermott H. Employment and the common cancers: Correlates of work ability during or following cancer treatment. Occup Med. 2009;59:381–9. doi:10.1093/occmed/kqp088

139. Munir F, Burrows J, Yarker J, Kalawsky K, Bains M. Women's perceptions of chemotherapy-induced cognitive side affects on work ability: A focus group study. J Clin Nurs. 2010; 19(9-10): 1362-70. doi: 10.1111/j.1365-2702.2009.03006.x (Table 1)

140. Munir F, Kalawsky K, Wallis D, Donaldson-Feilder E. Using intervention mapping to develop a work-related guidance tool for those affected by cancer. BMC Public Health 2013, 13:6 (13 pages) doi:10.1186/1471-2458-13-6

141. Musti MA, Collina N, Stivanello E, Bonfiglioli R, Giordani S, Morelli C, et al. [Perceived work ability at return to work in women treated for breast cancer: A questionnaire-based study.](https://pubmed-ncbi-nlm-nih-gov.proxy.kib.ki.se/30556532/) Med Lav. 2018;109(6):407-19. doi: 10.23749/mdl.v110i6.7241 (Table 1)

142. Newman RM. Re-defining one’s occupational self 2 years after breast cancer: A case study. WORK. 2013; 46(4): 439-44. DOI 10.3233/WOR-131679

143. Ng DWL, So SCY, Fielding R, Mehnert-Theuerkauf A, Kwong A, Suen D, et al. [Return to work, work productivity loss and activity impairment in Chinese breast cancer survivors 12-month post-surgery: a longitudinal study.](https://pubmed-ncbi-nlm-nih-gov.proxy.kib.ki.se/38463159/) Front Public Health. 2024;12:1340920. doi: 10.3389/fpubh.2024.1340920

144. Nieuwenhuijsen K, Bos-Ransdorp B, Uitterhoeve LL, Sprangers MA, Verbeek JH. Enhanced provider communication and patient education regarding return to work in cancer survivors following curative treatment: A pilot study. J Occup Rehabil. 2006;16(4):647-57. DOI 10.1007/s10926-006-9057-9

145. Nilsson M, Olsson M, Wennman-Larsen A, Petersson L-M, Alexanderson K. Return to work after breast cancer: Women’s experiences of encounters with different stakeholders. Eur J Oncol Nurs. 2011; 15(3):267-274. doi:10.1016/j.ejon.2011.03.005

146. Nilsson M, Petersson L, Wennman-Larsen A, Olsson M, Vaez M, Alexanderson K. Adjustment and social support at work early after breast cancer surgery and its associations with sickness absence. Psychooncology 2013;22: 2755–62. DOI: 10.1002/pon.3341

147. Nilsson M, Olsson M, Wennman-Larsen A, Petersson L, Alexanderson K. Women’s reflections and actions regarding working after breast cancer surgery – A focus group study. Psychooncology 2013;22: 1639–44. DOI: 10.1002/pon.3192 Although explicitly included in this study of women with breast cancer vis-à-vis return to work, no specific information can be gleaned about the women employed within the health care sector.

148. Ottati A, Feuerstein M. Brief self-report measure of work-related cognitive limitations in breast cancer survivors. J Cancer Surviv. 2013; 7:262–73. DOI 10.1007/s11764-013-0275-9

149. Peugniez C, Fantoni S, Leroyer A, Skrzypczak J, Duprey M, Bonneterre J. [Return to work after treatment for breast cancer: Single-center experience in a cohort of 273 patients.](https://pubmed-ncbi-nlm-nih-gov.proxy.kib.ki.se/20841323/) Ann Oncol. 2010;21(10):2124-5. doi: 10.1093/annonc/mdq556

150. Picard C, Agretelis J, DeMarco RF. Nurse experiences as cancer survivors: Part II—professional. Oncol Nurs Forum. 2004; 31(3) 537-41. DOI: 10.1188/04.ONF.537-542 (Table 2)

151. Porro B, Michel A, Zinzindohoué C, Bertrand P, Monrigal E, Trentini F, et al. [Quality of life, fatigue and changes therein as predictors of return to work during breast cancer treatment.](https://pubmed-ncbi-nlm-nih-gov.proxy.kib.ki.se/30664270/) Scand J Caring Sci. 2019;33(2):467-77. doi: 10.1111/scs.12646

152. Porro B, Campone M, Moreau P, Roquelaure Y. [Supporting the return to work of breast cancer survivors: From a theoretical to a clinical perspective.](https://pubmed-ncbi-nlm-nih-gov.proxy.kib.ki.se/35564514/) Int J Environ Res Public Health. 2022;19(9):5124. doi: 10.3390/ijerph19095124.

153. Pryce J, Munir F, Haslam C. Cancer survivorship and work: Symptoms, supervisor response, co-worker disclosure and work adjustment. J Occup Rehabil. 2007; 17:83–92. DOI 10.1007/s10926-006-9040-5

154. Pudrovska T, Carr D, McFarland M, Collins C. Higher-status occupations and breast cancer: A life-course stress Approach. Soc Sci Med. 2013; 89: 53–61. doi.org/10.1016/j.socscimed.2013.04.013

155. Reynolds F, Lim KH. Turning to art as a positive way of living with cancer: A qualitative study of personal motives and contextual influences. J Posit Psychol. 2007; 2:1, 66-75. DOI: 10.1080/17439760601083839

156. [Rick](https://pubmed-ncbi-nlm-nih-gov.proxy.kib.ki.se/?term=Rick+O&cauthor_id=33477192) O,  [Reuß-Borst](https://pubmed-ncbi-nlm-nih-gov.proxy.kib.ki.se/?term=Reu%C3%9F-Borst+M&cauthor_id=33477192) M, [Dauelsberg](https://pubmed-ncbi-nlm-nih-gov.proxy.kib.ki.se/?term=Dauelsberg+T&cauthor_id=33477192) T,  [Hass](https://pubmed-ncbi-nlm-nih-gov.proxy.kib.ki.se/?term=Hass+HG&cauthor_id=33477192) HG, [Volker König](https://pubmed-ncbi-nlm-nih-gov.proxy.kib.ki.se/?term=K%C3%B6nig+V&cauthor_id=33477192) V , [Reiner Caspari](https://pubmed-ncbi-nlm-nih-gov.proxy.kib.ki.se/?term=Caspari+R&cauthor_id=33477192) R, et al. Role of clinical, sociomedical and psychological factors on return to work of patients with breast cancer 6 months after rehabilitation. Rehabilitation (Stuttg) 2021;60(4):253-62. doi: 10.1055/a-1288-5824 GERMAN

157. Rosedale M. Survivor loneliness of women following breast cancer. Oncol Nurs Forum. 2009; 36(2): 175-83.

158. Ruggieri R, Gahanelli P, Belluccio C, Giorgi I, Fiabane E. Return to work after breast cancer: Barriers and facilitators. G Ital Med Lav Erg. 2021; 43(1): 66-73. ITALIAN

159. Sarfo MC, van Asselt KM, Frings-Dresen MH, de Jong F, van Dijk N, de Boer AGEM. [Views of breast cancer survivors on work participation guidance by general practitioners: A qualitative study.](https://pubmed-ncbi-nlm-nih-gov.proxy.kib.ki.se/35715735/) BMC Prim Care. 2022;23(1):152.  doi.org/10.1186/s12875-022-01768-x

160. Schlegel RJ, Manning M, Bettencourt B. Expectancy violations and the search for meaning among breast cancer survivors. J Posit Psychol. 2013; 8:5: 387-94. DOI: 10.1080/17439760.2013.807354

161. Schmidt ME; Scherer S, Wiskemann J, Steindorf K. Return to work after breast cancer: The role of treatment-related side effects and potential impact on quality of life. Eur J Cancer Care (Engl). 2019; 28 (4): 13051.

162. Şengün İnan F, Günüşen N, Özkul B, Aktürk N. [A dimension in recovery: Return to working life after breast cancer.](https://pubmed-ncbi-nlm-nih-gov.proxy.kib.ki.se/31651465/) Cancer Nurs. 2020;43(6):E328-34. DOI: 10.1097/NCC.0000000000000757 (Table 1)

163. Sharifi N, Ahmad S. [Breast cancer-related lymphedema: A critical review on recent progress.](https://pubmed-ncbi-nlm-nih-gov.proxy.kib.ki.se/39208532/) Surg Oncol. 2024;56:102124. doi: 10.1016/j.suronc.2024.102124

164. Sheppard DM, Frost D, Jefford M, O'Connor M, Halkett G. ['Beyond Cancer': A study protocol of a multimodal occupational rehabilitation programme to support breast cancer survivors to return work.](https://pubmed-ncbi-nlm-nih-gov.proxy.kib.ki.se/31843840/) BMJ Open. 2019;9(12):e032505. doi: 10.1136/bmjopen-2019-032505.

165. Shewbridge A, Wiseman T, Richardson A. Working while receiving chemotherapy: a survey of patients’ experiences and factors that influence these. Eur J Cancer Care 2012; 21(1): 117–23. DOI: 10.1111/j.1365-2354.2011.01304.x

166. Silver JK. A journey to make cancer rehabilitation the standard of care. Work. 2013; 46(4): 473-5. DOI 10.3233/WOR-131755 (Table 3)

167. Silver JK, Baima J, Newman R, Galantino ML, Shockney LD. Cancer rehabilitation may improve function in survivors and decrease the economic burden of cancer to individuals and society. Work. 2013; 46(4): 455-72. DOI 10.3233/WOR-131755

168. Silver JK. After cancer treatment: Heal faster, better, stronger. 2^nd^ ed. Baltimore: The Johns Hopkins University Press; 2015.

169. Singer S, Danker H, Roick J, Einenkel J, Briest S, Spieker H, et al. Effects of stepped psychooncological care on referral to psychosocial services and emotional well‐being in cancer patients: A cluster‐randomized phase III trial. Psychooncology. 2017;26:1675–83. DOI: 10.1002/pon.4492

170. Singer S, Roick J, Meixensberger J, Schiefke F, Brest S, Dietz A, et al. The effects of multi-disciplinary psycho-social care on socio-economic problems in cancer patients: a cluster-randomized trial. Supp Care Cancer. 2018;26(6):1851-9. doi.org/10.1007/s00520-017-4024-x

171. Skaczkowski G, Asahina A, Wilson C. Returning to work after cancer in Australia: What facilitates a positive return to work experience? J Occup Rehabil. 2021;31:41-9. doi.org/10.1007/s10926-020-09881-3

172. [Söderman](https://pubmed-ncbi-nlm-nih-gov.proxy.kib.ki.se/?term=S%C3%B6derman+M&cauthor_id=30255432) M , [E Friberg](https://pubmed-ncbi-nlm-nih-gov.proxy.kib.ki.se/?term=Friberg+E&cauthor_id=30255432) E, [K Alexanderson](https://pubmed-ncbi-nlm-nih-gov.proxy.kib.ki.se/?term=Alexanderson+K&cauthor_id=30255432) K , [A Wennman-Larsen](https://pubmed-ncbi-nlm-nih-gov.proxy.kib.ki.se/?term=Wennman-Larsen+A&cauthor_id=30255432) A  Women's experiences of encounters with healthcare professionals' regarding work after breast-cancer surgery and associations with sickness absence: a 2-year follow-up cohort study. Support Care Cancer. 2019;27(4):1197-206.  doi: 10.1007/s00520-018-4453-1

173. Soejima T, Kamibeppu K.  [Are cancer survivors well-performing workers? A systematic review.](https://pubmed-ncbi-nlm-nih-gov.proxy.kib.ki.se/27435320/) Asia Pac J Clin Oncol. 2016;12(4):e383-97. doi: 10.1111/ajco.12515

174. Stehle L, Hoosain M, van Niekerk L. A systematic review of work-related interventions for breast cancer survivors: Potential contribution of occupational therapists Work. 2022;72(1):59-73.  doi: 10.3233/WOR-210053

175. Stout NL, Santa Mina D, Lyons KD, Robb K, Silver JK. [A systematic review of rehabilitation and exercise recommendations in oncology guidelines.](https://pubmed-ncbi-nlm-nih-gov.proxy.kib.ki.se/33107982/) CA Cancer J Clin. 2021;71(2):149-75. doi: 10.3322/caac.21639

176. Sun W, Chen K, Terhaar A, Wiegmann DA, Heidrich SM, Tevaarwerk AJ, Sesto ME. [Work-related barriers, facilitators, and strategies of breast cancer survivors working during curative treatment.](https://pubmed-ncbi-nlm-nih-gov.proxy.kib.ki.se/28059814/) Work. 2016;55(4):783-95. doi: 10.3233/WOR-162449 (Table 1)

177. Sun Y, Armer JM. [A nurse's twenty-four-year journey with breast cancer-related lymphedema.](https://pubmed-ncbi-nlm-nih-gov.proxy.kib.ki.se/31033476/) Work. 2019;63(1):21-31. DOI:10.3233/WOR-192904 (Table 3)

178. Tachi T, Teramachi H, Tanaka K, Asano S, Osawa T, Kawashima A, et al. The impact of side effects from outpatient chemotherapy on presenteeism in breast cancer patients: A prospective analysis. Springerplus. 2016;5:327. doi: 10.1186/s40064-016-1979-x Although explicitly included in this study of women with breast cancer vis-à-vis return to work, no specific information can be gleaned about the woman who had been employed within the health care sector.

179. Tamminga S, de Boer A, Verbeek J, Frings-Dres MH. Breast cancer survivors’ views of factors that influence the return-to-work process-A qualitative study. Scand J Work Environ Health. 2012; 38(2): 144 –54. doi:10.5271/sjweh.3199 (Table 1)

180. Tamminga SJ, Verbeek JHAM, de Boer AGEM, van der Bij RM, Fings-Dresen MHW. A work-directed intervention to enhance the return to work of employees with cancer: A case study. Work. 2013; 46(4): 477–85. DOI 10.3233/WOR-131681

181. Tamminga SJ, Verbeek JH, Bos MM, Fons G, Kitzen JJ, Plaisier PW, et al. Effectiveness of a hospital-based work support intervention for female cancer patients—a multi-centre randomised controlled trial. PLoS One. 2013;8(5):e63271. doi:10.1371/journal.pone.0063271 Although explicitly included in this study of women with breast cancer vis-à-vis return to work, no specific information can be gleaned about the women employed within the health care sector.

182. Tamminga SJ, Verbeek J, Bos M, Fons G, Kitzen J, Plaisier PW, et al. Two-year follow-up of a multi-centre randomized controlled trial to study effectiveness of a hospital-based work support intervention for cancer patients. J Occup Rehab. 2019;29(4):701-10. doi.org/10.1007/s10926-019-09831-8 Although explicitly included in this study of women with breast cancer vis-à-vis return to work, no specific information can be gleaned about the women employed within the health care sector.

183. Tamura S, Sakaguchi K, Yamanaka R. Concerns and returns to work in patients with breast cancer receiving outpatient chemotherapy: A pilot study. Asia Pac J Oncol Nurs. 2019; 6 (2); 187-92. DOI 10.4103/apjon.apjon_58_18

184. Tan CJ, Yip SYC, Chan RJ, Chew L, Chan A. [Investigating how cancer-related symptoms influence work outcomes among cancer survivors: A systematic review.](https://pubmed-ncbi-nlm-nih-gov.proxy.kib.ki.se/34424498/) J Cancer Surviv. 2022;16(5):1065-78. doi: 10.1007/s11764-021-01097-5

185. Tan FL, Loh SY, Su TT, Veloo VW, Ng LL. [Return to work in multi-ethnic breast cancer survivors--A qualitative inquiry.](https://pubmed-ncbi-nlm-nih-gov.proxy.kib.ki.se/23317258/) Asian Pac J Cancer Prev. 2012;13(11):5791-7. doi: 10.7314/apjcp.2012.13.11.5791

186. Taskila T, de Boer A, van Dijk F, Verbeek J. Fatigue and its correlates in cancer patients who had returned to work—A cohort study. Psychooncology 2011; 20: 1236–41. DOI: 10.1002/pon.1843

187. Tavan H, Azadi A, Veisani Y. [Return to work in cancer patients: A Systematic Review and Meta-analysis.](https://pubmed-ncbi-nlm-nih-gov.proxy.kib.ki.se/30820118/) Indian J Palliat Care. 2019;25(1):147-52. doi: 10.4103/IJPC.IJPC_114_18

188. Tay MRJ, Wong CJ, Aw HZ.  [Return to work in breast cancer patients following an interdisciplinary rehabilitation program in a community-based cancer rehabilitation center: A pilot study.](https://pubmed-ncbi-nlm-nih-gov.proxy.kib.ki.se/38610227/) Healthcare (Basel). 2024;12(7):805. doi: 10.3390/healthcare12070805

189. Thijs KM, de Boer AGEM, Vreugdenhil G, van de Wouw AJ, Houterman S, Schep G. Rehabilitation using high-intensity physical training and long-term return-to-work in cancer survivors. J Occup Rehabil. 2012; 22(2):220-9. DOI 10.1007/s10926-011-9341-1

190. Thomas-Maclean R, Towers A, Quinlan E, Hack TF, Kwan W, Miedema B, Tilley A, Graham P.  ["This is a kind of betrayal": A qualitative study of disability after breast cancer.](https://pubmed-ncbi-nlm-nih-gov.proxy.kib.ki.se/19526082/) Curr Oncol. 2009;16(3):26-32. doi: 10.3747/co.v16i3.389 Although explicitly included in this study of women with breast cancer vis-à-vis return to work, no specific information can be gleaned about the women employed within the health care sector.

191. Thompson Buum HA. [Sharing my diagnosis: How much is too much?](https://pubmed-ncbi-nlm-nih-gov.proxy.kib.ki.se/30858262/) Ann Fam Med. 2019;17(2):173-5. doi: 10.1370/afm.2365 (Table 3)

192. Thompson Buum HA.  [Out of the mouths of babes: A physician discusses her cancer diagnosis with her two young children.](https://pubmed-ncbi-nlm-nih-gov.proxy.kib.ki.se/30433851/) J Clin Oncol. 2019;37(1):81-3. doi: 10.1200/JCO.18.00989 (Table 3)

193. Tiedtke C, de Rijk A, de Casterlé BD, Christiaens M-R, Donceel P. Experiences and concerns about 'returning to work' for women breast cancer survivors: A literature review. Psychooncology, 2010; 19(7): 677-83. DOI: 10.1002/pon.1633

194. Tiedtke C, de Rijk A, Donceel P, Christiaens M, Dierckx de Casterlé B. Survived but feeling vulnerable and insecure: a qualitative study of the mental preparation for RTW after breast cancer treatment. BMC Public Health 2012; 12:538 (13 pages) doi:10.1186/1471-2458-12-538 Although explicitly included in this study of women with breast cancer vis-à-vis return to work, no specific information can be gleaned about the women employed within the health care sector.

195. Tiedtke C, Donceel P, Knops L, Désiron H, de Casterlé BD, de Rijk A, Supporting return-to-work in the face of legislation: Stakeholders’ experiences with return-to-work after breast cancer in Belgium. J Occup Rehab. 2012; 22(2): 241-51. DOI 10.1007/s10926-011-9342-0

196. Tiedtke C, Donceel P, de Rijk A, de Casterlé B. J Occup Rehab. Return to work following breast cancer treatment: The employers’ side. 2014; 24(3): 399-409**.** DOI 10.1007/s10926-013-9465-6

197. Tiedtke C, de Casterlé B, Donceel P, de Rijk A. Workplace support after breast cancer treatment: Recognition of vulnerability. Disabil Rehabil. 2015; 37(19): 1770. DOI: 10.3109/09638288.2014.982830 Although explicitly included in this study of women with breast cancer vis-à-vis return to work, no specific information can be gleaned about the women employed as caregivers.

198. Tiedtke CM, Dierckx de Casterle´ B, Frings-Dresen MHW, De Boer AGEM, Greidanus MA, Tamminga SJ et al. Employers’ experience of employees with cancer: trajectories of complex communication. J Cancer Surv. 2017;11: 562-77. DOI 10.1007/s11764-017-0626-z

199. Tiedtke CM, Blonk RWB, Van Rhenen W , Van Egmond M, Joosen MCW. Tailored support for preparing employees with cancer to return to work: Recognition and gaining new insights in an open atmosphere. Work. 2023;76(4):1519-33. DOI:10.3233/WOR-220566 Although explicitly included in this study of women with breast cancer vis-à-vis return to work, no specific information can be gleaned about the women employed within the health care sector.

200. Tighe M, Molassiotis A, Morris J , Richardson J. Coping, meaning and symptom experience: A narrative approach to the overwhelming impacts of breast cancer in the first year following diagnosis. Eur J Oncol Nurs. 2011; 15: 226-32. doi:10.1016/j.ejon.2011.03.004 (Table 1)

201. Timperi A, Ergas I, Rehkopf D, Roh J, Kwan M, Kushi L. Employment status and quality of life in recently diagnosed breast cancer survivors. Psychooncology. 2013; 22:1411-20. DOI: 10.1002/pon.3157

202. Torp S, Nielsen R, Gudbergsson SB, Dahl A. Worksite adjustments and work ability among employed cancer survivors. Support Care Cancer. 2012; 20(9): 149-56. DOI 10.1007/s00520-011-1325-3

203. van Maarschalkerweerd PEA, Schaapveld M, Paalman CH, Aaronson NK, Duijts SFA. [Changes in employment status, barriers to, and facilitators of (return to) work in breast cancer survivors 5-10 years after diagnosis.](https://pubmed-ncbi-nlm-nih-gov.proxy.kib.ki.se/30907148/) Disabil Rehabil. 2020;42(21):3052-8. doi: 10.1080/09638288.2019.1583779 (Table 1)

204. Van Muijen P, Weevers N, Snels I, Duijts S, Bruinvels D, Schellart A, et al. Predictors of return to work and employment in cancer survivors: A systematic review. Eur J Cancer Care. 2013; 22, 144–60. DOI: 10.1111/ecc.12033

205. Vayr F, Montastruc M, Savall F, Despas F, Judic E, Basso M, et al. [Work adjustments and employment among breast cancer survivors: A French prospective study.](https://pubmed-ncbi-nlm-nih-gov.proxy.kib.ki.se/31001691/) Support Care Cancer. 2020;28(1):185-92.  doi.org/10.1007/s00520-019-04799-w

206. Vignes S, Fau-Prudhomot P, Simon L, Sanchez-Bréchot ML, Arrault M, Locher F. [Impact of breast cancer-related lymphedema on working women.](https://pubmed-ncbi-nlm-nih-gov.proxy.kib.ki.se/30982096/) Support Care Cancer. 2020;28(1):79-85. doi: 10.1007/s00520-019-04804-2 (Table 1)

207. Wells M, Williams B, Firnigl D, Lang H, Coyle J, Kroll T et al. Supporting ‘work-related goals’ rather than ‘return to work’ after cancer? A systematic review and meta-synthesis of 25 qualitative studies. Psychooncology 2013; 22: 1208–19. DOI: 10.1002/pon.3148

208. Yagil D, Cohen M. The role of perceived supervisor resistance in successful work sustainability among cancer survivors. Work. 2022; 73 (1): 203–10. DOI:10.3233/WOR-205341

209. You KL, Cummings MH, Bender CM, Fennimore LA, Rosenzweig MQ, Dierkes AM, et al. A scoping review on work experiences of nurses after being diagnosed with cancer. Oncol Nurs Forum. 2023; 50(6): 714–24. [doi.org/10.1188/23.ONF.714-724](https://doi.org/10.1188/23.ONF.714-724) **Helped identify several of the studies included in Tables 2 and 3**

210. You KL, Wang Y, Zhang Y, Bender CM, Fennimore LA, Rosenzweig MQ, et al. [Nurses' returning to work after cancer: A focus group study.](https://pubmed-ncbi-nlm-nih-gov.proxy.kib.ki.se/39388798/) Nurs Outlook. 2024;72(6):102290. doi: 10.1016/j.outlook.2024.102290 (Table 2)

211. Zamanzadeh V, Valizadeh L, Zirak M*,* Rahmani A. Taking an obscure path, a common concern during returning to work after cancer. J Vocat Rehabil. 2019; 51 (3): 369-76. DOI:10.3233/JVR-191052 (Table 1)

212. Zamanzadeh V, Valizadeh L, Rahmani A, Zirak M, Desiron H. Cancer survivors' experiences of return to work: A qualitative study. Psychooncology. 2018; 27(10):2398–404. [doi.org/10.1002/pon.4840](https://doi.org/10.1002/pon.4840) (Table 1)

213. Zavrtanik Čelan A, Prosen M. Nurses' return to active work after breast cancer treatment: A descriptive qualitative study. Onkologija. 2022; 26(2): 6-14. SLOVENIAN (Table 2)

214. Zegers AD, van Belzen M, Engelen V, Richel C, Dona DJS, van der Beek AJ, Duijts SFA. Cancer survivors' experiences with conversations about work‐related issues in the hospital setting. Psychooncology. 2021; 30(1): 27-34. DOI: 10.1002/pon.5529

215. Zhang XJ, Zhou L. Cochrane review summary for cancer nursing: Interventions to enhance return to work for cancer patients. Cancer Nurs. 2013; 36(1): 4-5. DOI: 10.1097/NCC.0b013e318277b564

216. Zhu XL, Lei JJ, Chen R, Chen Z, Xiong Z, Yang L, et al. Cancer recurrence fear and return to work in breast cancer survivors:The mediating effects of health literacy J Multidiscip Healthc. 2025; 18: 1031-41.

217. Zomkowski K, Cruz de Souza B, Pinheiro da Silva F, Moreira GM, de Souza Cunha N, Sperandio FF. [Physical symptoms and working performance in female breast cancer survivors: a systematic review.](https://pubmed-ncbi-nlm-nih-gov.proxy.kib.ki.se/28325132/) Disabil Rehabil. 2018;40(13):1485-93. doi: 10.1080/09638288.2017.1300950

218. Zomkowski K, de Souza BC, Moreira GM, Volkmer C, da Silva Honório GJ, Moraes Santos G, et al. Qualitative study of return to work following breast cancer treatment. Occup Med (Oxford). 2019; 69(3):189-94. doi:10.1093/occmed/kqz024
